# The Y172 Monoclonal Antibody Against p-c-Jun (Ser63) Is a Marker of the Postsynaptic Compartment of C-Type Cholinergic Afferent Synapses on Motoneurons

**DOI:** 10.3389/fncel.2019.00582

**Published:** 2020-01-24

**Authors:** Alaó Gatius, Olga Tarabal, Paula Cayuela, Anna Casanovas, Lídia Piedrafita, Sara Salvany, Sara Hernández, Rosa M. Soler, Josep E. Esquerda, Jordi Calderó

**Affiliations:** ^1^Unitat de Neurobiologia Cel·lular, Departament de Medicina Experimental, Facultat de Medicina, Universitat de Lleida and Institut de Recerca Biomèdica de Lleida (IRBLleida), Lleida, Spain; ^2^Unitat de Senyalització Neuronal, Departament de Medicina Experimental, Facultat de Medicina, Universitat de Lleida and Institut de Recerca Biomèdica de Lleida (IRBLleida), Lleida, Spain

**Keywords:** motoneuron, C-bouton, phospho-c-Jun, Y172 antibody, endoplasmic reticulum-plasma membrane contacts

## Abstract

C-bouton-type cholinergic afferents exert an important function in controlling motoneuron (MN) excitability. During the immunocytochemical analysis of the role of c-Jun in MNs with a monoclonal (clone Y172) antibody against phospho (p)-c-Jun (serine [Ser]63), unexpected labeling was identified in the cell body cytoplasm. As predicted for c-Jun in adult spinal cord, very few, if any MNs exhibited nuclear immunoreactivity with the Y172 antibody; conversely, virtually all MNs displayed strong Y172 immunostaining in cytoplasmic structures scattered throughout the soma and proximal dendrites. The majority of these cytoplasmic Y172-positive profiles was closely associated with VAChT-positive C-boutons, but not with other types of nerve afferents contacting MNs. Ultrastructural analysis revealed that cytoplasmic Y172 immunostaining was selectively located at the subsurface cistern (SSC) of C-boutons and also in the inner areas of the endoplasmic reticulum (ER). We also described changes in cytoplasmic Y172 immunoreactivity in injured and degenerating MNs. Moreover, we noticed that MNs from NRG1 type III-overexpressing transgenic mice, which show abnormally expanded SSCs, exhibited an increase in the density and size of peripherally located Y172-positive profiles. A similar immunocytochemical pattern to that of the Y172 antibody in MNs was found with a polyclonal antibody against p-c-Jun (Ser63) but not with another polyclonal antibody that recognizes c-Jun phosphorylated at a different site. No differential band patterns were found by western blotting with any of the antibodies against c-Jun or p-c-Jun used in our study. In cultured MNs, Y172-positive oval profiles were distributed in the cell body and proximal dendrites. The *in vitro* lentiviral-based knockdown of c-Jun resulted in a dramatic decrease in nuclear Y172 immunostaining in MNs without any reduction in the density of cytoplasmic Y172-positive profiles, suggesting that the synaptic antigen recognized by the antibody corresponds to a C-bouton-specific protein other than p-c-Jun. Our results lay the foundation for further studies aimed at identifying this protein and determining its role in this particular type of synapse.

## Introduction

Motor behavior is mediated by the voluntary contraction of skeletal muscles, which are innervated by the distinct brainstem and spinal cord motoneurons (MNs). In this regard, the execution of complex movements, such as those involved in locomotion, requires the cooperation and refined coordination of different muscles, whose activation is modulated by intricate neuronal circuits in the spinal cord. MNs play a crucial role in these neuromodulatory circuits by adjusting their final motor output and ensuring the accuracy of the movement. MN activity is controlled by excitatory (mainly glutamatergic and cholinergic) and inhibitory (mainly GABAergic and glycinergic) inputs with supraspinal and intraspinal origins (Grillner, 2006; Jordan et al., 2008). Cholinergic inputs are essentially represented by so-called C-boutons, a type of synaptic terminal that contacts the soma and proximal dendrites of α-MNs located in the spinal cord and in cranial motor nuclei except those that innervate extrinsic eye muscles (Conradi and Skoglund, 1969; Connaughton et al., 1986; Davidoff and Irintchev, 1986; Nagy et al., 1993; Hellström et al., 1999, 2003; Deardorff et al., 2014; Witts et al., 2014; Rozani et al., 2019). C-boutons are large nerve terminals (3–6 μm in diameter in humans and 1–8 μm in rodents) that contain many densely packed clear spherical or slightly flattened synaptic vesicles (Deardorff et al., 2014). The postsynaptic counterpart of C-boutons exhibits a unique and highly specialized structure, of non-well-defined function, termed subsurface cistern (SSC), which is closely apposed to the MN plasma membrane. In fact, SSC is a 10–15 nm-wide and flattened lamella-like arrangement of the rough endoplasmic reticulum (ER) located 5–8 nm below the postsynaptic membrane (Conradi, 1969). Acetylcholine (ACh), released by C-boutons as a neurotransmitter, binds to metabotropic M2 muscarinic receptors postsynaptically located on MNs (Hellström et al., 2003). Immunocytochemical studies have revealed other molecules in addition to clusters of M2 muscarinic receptors in the postsynaptic compartments of C-boutons; for example Kv2.1 voltage-gated K^+^ channels (Muennich and Fyffe, 2004), Ca^2+^-activated K^+^ (SK) channels (Deardorff et al., 2013), N-type Ca^+^ channels (Wilson et al., 2004), vesicle-associated membrane protein 2 (VAMP-2; Hellström et al., 2003), sigma-1 receptors (S1Rs; Mavlyutov et al., 2012, 2013) and neuregulin-1 (NRG1; Gallart-Palau et al., 2014; Casanovas et al., 2017; Salvany et al., 2019) are located postsynaptically at C-bouton synapses; among these molecules, S1R, Kv2.1 and NRG1 accumulate within the SSC in segregated regions, which probably represent distinct spatial domains with high specificity (Gallart-Palau et al., 2014; Casanovas et al., 2017; Salvany et al., 2019). In the lumbar spinal cord, C-bouton inputs arise from a small cluster of cholinergic interneurons (V0_C_ interneurons) that express the paired transcription factor *Pitx2* and are located lateral to the central canal (Zagoraiou et al., 2009). It has been reported that the majority (~70%) of the V0_C_ interneurons project to ipsilateral spinal MNs, whereas the remaining V0_C_ cells project to MNs located either on the contralateral side or on both sides of the spinal cord (Miles et al., 2007). Studies performed in mice have demonstrated that V0_c_ and C-boutons have an important role in the activation of specific hind-limb muscles through the regulation of MN output in a task-dependent manner (Zagoraiou et al., 2009). Thus, the neuromodulatory activity of C-boutons appears to be critical in controlling the excitation state of MNs, likely through reducing outward K^+^ currents and, therefore, regulating after-hyperpolarization (AHP) potentials (Miles et al., 2007; Deardorff et al., 2014; Witts et al., 2014). In this regard, C-boutons would determine the differences in intrinsic cellular excitability found in distinct MN subtypes; MNs that innervate slow-twitch muscles (slow [S] MNs) have a longer AHP latency after an action potential and, consequently, a higher excitability and a slower firing rate than that of MNs that innervate fast-twitch muscles [fast-fatigable (FF MNs; for reviews, see Roselli and Caroni, 2015; Nijssen et al., 2017]. It has been suggested that the intrinsic hyperexcitability of S MNs confers resistance to degeneration, while FF MNs are particularly vulnerable in MN diseases such as amyotrophic lateral sclerosis (ALS) and spinal muscular atrophy (SMA; Saxena et al., 2013; Roselli and Caroni, 2015; Arumugam et al., 2017). Moreover, it has been shown that the density of C-boutons is higher on FF MNs than on S MNs (Hellström et al., 2003; Casanovas et al., 2017), and changes in this type of synapse have been reported in murine models of ALS (SOD1^G93A^ mice) and SMA (SMNΔ7 and *Smn*^2B/-^ mice) throughout the disease course (Pullen and Athanasiou, 2009; Saxena et al., 2013; Gallart-Palau et al., 2014; Milan et al., 2015; Cerveró et al., 2018).

The transcription factor c-Jun is a component of the AP-1 complex, and it has been reported to have a double function as a regulator of both the death and survival (protection-regeneration) of neurons (Herdegen et al., 1997). Thus, c-Jun has been shown to participate in different processes, including cell cycle regulation, cellular differentiation, organogenesis, apoptosis and tumor transformation (Herdegen et al., 1997). The phosphorylation of c-Jun at serine (Ser) 63 and/or 73, a process mediated by c-Jun N-terminal kinases (JNKs), strongly potentiates the capability of this factor to activate gene transcription (Smeal et al., 1991, 1992, 1994; Waetzig et al., 2006; Haeusgen et al., 2009). In this regard, it has been shown that JNKs are rapidly activated, promoting the increase of phospho (p)-c-Jun levels following peripheral nerve injury or neuronal stress in spinal MNs of neonatal and adult rats (Waetzig et al., 2006; Yuan et al., 2010).

In immunocytochemical experiments performed with a phospho-specific monoclonal, clone Y172, antibody against c-Jun to examine its role in MN degeneration, we identified unexpected cytoplasmic immunolabeling. Y172 antibody was hosted in the rabbit and specifically detects c-Jun phosphorylated on Ser63. Upon the application of this antibody to the spinal cord from adult mice, nuclear immunoreactivity was found in very few, if any, MNs. In contrast, the vast majority of MNs exhibited prominent Y172 immunostaining in the form of profiles widely distributed in the cytoplasm. Some of these profiles were adjacent to the plasma membrane of the cell body and proximal dendrites. This pattern of immunostaining was only observed in large-sized MNs, presumably α-MNs, but not in smaller MNs (γ-MNs) or interneurons. Due to the restricted location of cytoplasmic Y172 immunoreactivity in α-MNs and the synaptic-like appearance of Y172-positive profiles located peripherally in cell bodies, we examined their relationship with axon terminals contacting α-MNs and found that Y172 immunoreactive profiles were closely associated with C-boutons. This finding prompted us to analyze in-depth the significance of the unusual immunoreactivity of this antibody against the transcription factor c-Jun found in MNs and the role that its association with C-boutons plays in cholinergic synapse development and maintenance. We examined the compartmentation of Y172 immunoreactivity and its subcellular location in MNs. We analyzed the changes in motoneuronal Y172-positive structures after peripheral nerve injury and in murine models of ALS and SMA. We compared Y172 immunolabeling in the spinal cord with that of other polyclonal antibodies against p-c-Jun. Additionally, we analyzed Y172 immunostaining *in vitro* in NSC-34 cells and spinal cord MNs, in which we used RNA interference to knock down c-Jun.

## Materials and Methods

### Animals and Surgical Procedures

All animal experimentation procedures were performed according to the European Committee Council Directive and the norms established by the *Generalitat de Catalunya* [published as a law in the *Diari Oficial de la Generalitat de Catalunya* (DOGC) 2073, 1995]. All experiments were previously evaluated and approved by the Committee for Animal Care and Use of our university. Mice were housed in the University of Lleida Animal Care Facility on a 12-h light/dark cycle with ad libitum access to standard laboratory chow and water.

Several transgenic mouse lines were used in this study. SOD1^G93A^ (B6SJL-Tg(SOD1-G93A)1Gur/J) mice were purchased from Jackson Laboratory (Sacramento, CA, USA) and maintained as hemizygotes by breeding transgenic males with C57BL/6 females. *Smn*^2B/-^ mice were obtained by crossing heterozygote male *Smn* gene knockout mice (*Smn*^+/–^; B6.Cg-Smn1^tm2Mrph^/J, stock 007963; Jackson Laboratory, Bar Harbor, ME, USA) with homozygote females with the 2B mutation (*Smn*^2B/2B^; a kind gift from Dr. Rashmi Kothary, University of Ottawa, Ottawa, ON, Canada; DiDonato et al., 2001); the progeny of these transgenic mouse lines were identified by PCR genotyping of DNA extracted from the tail by using specific primers as described previously (Schrank et al., 1997; Cerveró et al., 2018). Aged-matched wild-type (WT) littermates of the transgenic animals were used as controls. Moreover, transgenic mice overexpressing HA-tagged full-length NRG1 type III (HA-NRG1^FL^ type III, hereafter referred to as NRG1-III; Michailov et al., 2004; Velanac et al., 2012) were also used. For experiments in normal mice, nontransgenic CD1 animals (purchased from Harlan Laboratories, Castellar del Vallès, Barcelona, Catalonia, Spain) of different ages were used. Additionally, adult rats [Sprague–Dawley, postnatal day (P) 60] and chick embryos [*Hy-line white*, embryonic day (E) 20] obtained from fertilized eggs (Avigan Terralta, Vinallop, Catalonia, Spain) incubated in the laboratory were also used. The age of the chick embryos was determined by reference to the Hamburger-Hamilton stage series (Hamburger and Hamilton, 1992).

A group of CD1 mice was subjected to unilateral sciatic nerve transection on P60. For this procedure, animals were anesthetized with a combination of ketamine (100 mg/kg) and xylazine (10 mg/kg), and the sciatic nerve at the femoral level was exposed and transected. The proximal stump of the transected nerve was ligated to prevent spontaneous reinnervation. Postoperative analgesia was achieved by an intraperitoneal (i.p.) injection of buprenorphine (0.05 mg/kg).

### NSC-34 Cell Culture and Differentiation

The spinal cord × neuroblastoma hybrid cell line (NSC-34) was obtained from CELLutions Biosystems Inc. (Cedarlane Laboratories, Burlington; ON, Canada). NSC-34 cells were maintained in a proliferation medium consisting of Dulbecco’s modified Eagle’s medium (DMEM, Gibco, Waltham, MA, USA) supplemented with 10% fetal bovine serum (FBS, Gibco, Waltham, MA, USA), 0.25% penicillin/streptomycin solution (Sigma-Aldrich, Saint Louis, MO, USA) and 2% 200 mM L-glutamine (Gibco, Waltham, MA, USA). Cells were subcultured every 2–3 days.

For differentiation, cells were seeded in collagen (Sigma-Aldrich, Saint Louis, MO, USA)-coated plates. A total of 12,000 NSC-34 cells/well were plated in 4-well culture dishes containing collagen-coated round glass coverslips for immunocytochemical analysis, and 120,000 NSC-34 cells/well were plated in 6-well collagen-coated culture dishes for western blot analysis. Twenty-four hours after seeding, the proliferation medium was exchanged for fresh differentiation medium containing 1:1 DMEM/Ham’s F12 (Gibco, Waltham, MA, USA), 1% FBS (Gibco, Waltham, MA, USA), 1% modified Eagle’s medium nonessential amino acids (MEM NEAA, Gibco, Waltham, MA, USA) and 1% penicillin/streptomycin (Sigma-Aldrich, Saint Louis, MO, USA). Cells were maintained in the presence or absence of retinoic acid (RA, 1 μM) in differentiation medium, as previously described (Johann et al., 2011; Maier et al., 2013; Madji Hounoum et al., 2016). The differentiation medium was changed every 2 days. NSC-34 cells were allowed to differentiate for up to 8 days to identify the optimal differentiation state. Following differentiation, two morphologically distinct cell populations, namely, undifferentiated round-shaped cells with small processes and cells with a more differentiated appearance and long branches that resembled MNs, were observed. Every 2 days (2, 4, 6 and 8 days following differentiation), cell samples were taken and processed for immunocytochemistry and western blot analysis.

### Spinal Cord MN Cultures

Purified primary cultures of spinal cord MNs from E13 CD1 mice were prepared as previously described (Arce et al., 1999; Gou-Fabregas et al., 2009) with some minor modifications. Briefly, isolated MNs were plated in 4-well tissue culture dishes coated with polyornithine/laminin (Sigma-Aldrich, Saint Louis, MO, USA) for immunocytochemistry (20,000 cells/well) and western blot analysis (50,000 cells/well). The culture medium used was neurobasal medium (Gibco, Waltham, MA, USA) supplemented with B27 (2% v/v, Gibco, Waltham, MA, USA), horse serum (2% v/v, Thermo Fisher Scientific, Waltham, MA, USA), L-glutamine (0.5 mM, Gibco, Waltham, MA, USA), 2-mercaptoethanol (25 μM, Sigma-Aldrich, Saint Louis, MO, USA), and a recombinant neurotrophic factor cocktail containing brain-derived neurotrophic factor (BDNF, 1 ng/ml), glial cell line-derived neurotrophic factor (GDNF, 10 ng/ml), ciliary neurotrophic factor (CNTF, 10 ng/ml), cardiotrophin-1 (CT-1, 10 ng/ml), and hepatocyte growth factor (HGF, 10 ng/ml; Gibco, Waltham, MA, USA). Twenty hours after plating, aphidicolin (2 μg/ml, Sigma-Aldrich, Saint Louis, MO, USA) was added to the culture medium of some dishes and maintained throughout the experiment. MNs were collected 3, 6 and 12 days after seeding and processed for immunocytochemistry as well as protein extraction.

Mixed primary cultures of the spinal cord from E13 CD1 mice were prepared as previously described (Roy et al., 1998). Briefly, dissociated cells were plated at a density of 300,000 per well in 4-well Nunclon Delta (Thermo Fisher Scientific, Waltham, MA, USA) culture dishes containing round glass coverslips coated with a poly-D-lysine plus Matrigel basement membrane matrix (Corning, Bedford, MA, USA). The cells were then maintained in minimum essential medium (Gibco, Waltham, MA, USA) enriched with 5 g/l glucose and supplemented with 3% horse serum, 10 ng/ml nerve growth factor and B27 medium (Gibco, Waltham, MA, USA). On day 6, the cultures were treated with 1.4 μg/ml cytosine-β-arabinoside (Sigma-Aldrich, Saint Louis, MO, USA) to minimize the growth of nonneuronal cells. Cultures kept for 22–25 days *in vitro* were processed for immunocytochemistry.

### Plasmids and the Production of Lentiviral Particles

For gene silencing experiments, small hairpin RNA (shRNA) molecules were used. Constructs were generated in pSUPER.retro.puro (Sigma-Aldrich, Saint Louis, MO, USA) using specific oligonucleotides (Invitrogen, Waltham, MA, USA) targeting the c-Jun sequence (indicated by capital letters) as follows: shRNA A6, gatccccAAAGGAAGCTGGAGCGGATttcaagagaATCCGCTCCAGCTTCCTTTttttt (forward) and agctaaaaaAAAGGAAGCTGGAGCGGATtctcttgaaATCCGCTCCAGCTTCCTTTggg (reverse; targeting the 1715-bp region of the c-Jun mRNA sequence); shRNA B4, gatccccAAGTCATGAACCACGTTAAttcaagagaTTAACGTGGTTCATGACTTttttt (forward) and agctaaaaaAAGTCATGAACCACGTTAAtctcttgaaTTAACGTGGTTCATGACTTggg (reverse; targeting the 1874-bp region of the c-Jun mRNA sequence); and shRNA C2, gatccccATCCGTTTGTCTTCATTTTttcaagagaAAAATGAAGACAAACGGATttttt (forward) and agctaaaaaATCCGTTTGTCTTCATTTTtctcttgaaAAAATGAAGACAAACGGATggg (reverse) targeting the 150-bp region of the c-Jun mRNA sequence.

Adaptors for cloning the oligonucleotides into the BglII/HindII sites of p-SUPER.retro.puro were added as required. *Escherichia coli* (*E. coli*) DH5 cells were transformed with the plasmid. When colonies appeared, they were selected, sequenced to verify that they contained the desired sequence, and purified. Lentiviral constructs were generated by digesting pSUPER-sh with EcoRI and ClaI restriction enzymes to replace the H1 promoter with the H1-short hairpin RNA (shRNA) cassette in the pLVTHM lentiviral plasmid. The pLVTHM vector contained green fluorescent protein (GFP) under the control of an EF-1 alpha promoter for monitoring transduction efficiency. The production of lentiviral particles was carried out as previously described (Garcera et al., 2011). Lentiviruses were propagated in human embryonic kidney 293T (HEK293T) cells using the polyethyleneimine (Sigma-Aldrich, Saint Louis, MO, USA) cell transfection method. Briefly, 20 μg of either pLVHM-A6, pLVTHM-B4, pLVTHM-C2 or pLVTHM-EV (empty vector, used as a control; hereafter referred to as EV), 13 μg of pSPAX2, and 7 μg of pMD2G were transfected into HEK293T cultures (Zufferey et al., 1997). Cells were allowed to produce lentivirus for 72 h. Then, the medium was centrifuged at 1,000 rpm for 10 min, and the supernatant was filtered using a 22-μm filter. The medium containing the lentiviruses was stored at −80°C. A 12-well dish plated with 20,000 HEK293T cells per well was used to determine the biological titers of viral preparations, which were expressed as the number of transducing units per ml (TU/ml). After 48 h, the percentage of GFP-positive cells was measured, and viruses were used for the experiments at concentrations of 4 × 10^5^–1 × 10^6^ TU/ml. For lentiviral transduction, MNs isolated from the spinal cord of E13 CD1 mice were plated in 4-well dishes, as indicated above. Four hours after seeding, medium containing lentivirus (2 TU/cell) was added. The medium was changed 24 h later, and the infection efficiency of each experiment was determined by directly counting the GFP-positive cells. The vast majority of cells were GFP positive, with a greater than 90% cell transduction frequency (EV: 94.96% ± 0.50; A6: 96.63% ± 1.52; B4: 96.28% ± 1.79; and C2: 93.89% ± 4.42: *p* > 0.05, one-way ANOVA, *post hoc* Bonferroni’s test, 3 different experiments). After 6 days *in vitro* (DIV), cells were collected and processed for immunocytochemistry and western blot analysis.

### Immunocytochemistry and Imaging

Spinal cords, brains and brainstems were obtained from anesthetized mice that were transcardially perfused with 4% paraformaldehyde (PF) in 0.1 M phosphate buffer (PB), pH 7.4. Spinal cord samples were postfixed overnight in the same fixative at 4°C and then cryoprotected with 30% sucrose in 0.1 M PB containing 0.02% sodium azide. Serial transverse cryostat sections (16-μm thick) of the lumbar region of the spinal cord were collected on gelatin-coated glass slides and stored at −80°C. In some experiments, transverse spinal cord sections (200-μm thick) were obtained with a vibratome, collected in 0.1 M PB and transferred to a drop of PB placed on Fisherbrand Superfrost Plus microscope slides (Thermo Fisher Scientific, Waltham, MA, USA). A coverslip was then applied to the tissue, which was squashed with forceps. The preparations were then frozen in liquid N_2,_ and after the coverslip was removed, slides with retained tissue were fixed in methanol at −20°C for 10 min. Serial coronal sections (80-μm thick) of the whole brain and brainstem were also obtained with a vibratome and collected in 0.1 M PB. Cultured cells plated on glass coverslips were fixed with 4% PF in 0.1 M PB, pH 7.4 for 1 h at 4°C. After washing with phosphate-buffered saline (PBS), immunocytochemistry was performed as described below.

For immunocytochemistry, tissue sections or cultured cells were permeabilized with PBS containing 0.1% Triton X-100 for 30 min, blocked with either 10% normal goat serum or normal horse serum in PBS for 1 h at room temperature, and then incubated overnight at 4°C with an appropriate primary antibody mixture. The primary antibodies used were rabbit monoclonal anti-phospho-c-Jun (serine [Ser]63) clone Y172 (diluted 1:300, hereafter referred to as the Y172 antibody; Abcam, Cambridge, UK; cat. ab32385 or Millipore, Burlington, MA, USA; cat.# 04-212); rabbit polyclonal anti-phospho-c-Jun (Ser63; 1:100; Cell Signaling, Danvers, MA, USA; cat.# 9261); rabbit polyclonal anti-phospho-c-Jun (Ser73; 1:100; Cell Signaling; cat.# 9164); guinea pig polyclonal anti-synaptophysin 1 (1:500; Synaptic Systems, Goettingen, Germany; cat.# 101004); guinea pig polyclonal anti-vesicular acetylcholine transporter (VAChT; 1:500; Synaptic Systems, Goettingen, Germany; cat.# 139105); guinea pig polyclonal anti-vesicular glutamate transporter 1 (VGluT1, 1:500; Synaptic Systems, Goettingen, Germany; cat.# 135304); guinea pig polyclonal anti-vesicular GABA transporter (VGAT, 1:200; Synaptic Systems, Goettingen, Germany; cat.# 131004); mouse monoclonal anti-synaptic vesicle glycoprotein 2A (SV2, 1:1,000; Developmental Studies Hybridoma Bank, Iowa City, IA, USA; cat.# AB_2315386); mouse monoclonal anti-sigma-1 receptor (S1R, 1:50; Santa Cruz Biotechnology, Dallas, TX, USA; cat.# sc-137075); mouse monoclonal anti-Kv2.1 voltage-gated potassium channel (Kv2.1, 1:100; NeuroMab, Davis, CA, USA; cat.# 73-014); sheep polyclonal anti-choline acetyltransferase (ChAT, 1:1,000; Abcam cat.# Ab18736); rabbit polyclonal anti-ChAT (1:200; Millipore, Burlington, MA, USA; cat.# AB143); rabbit polyclonal anti-neuregulin-1 (NRG1) type III (extracellular, 1:250; Alomone labs, Jerusalem, Israel, cat.# ANR 113); mouse monoclonal anti-NRG-CRD, type III, clone N126B/31 (1:250; Millipore; cat.# MABN534); rabbit polyclonal anti-NRG1 1 α/β 1/2 (1:300; Santa Cruz Biotechnology, Dallas, TX, USA; cat.# sc-348); mouse monoclonal anti-Golgi matrix protein of 130 kDa (GM130, 1:200; BD Biosciences, San Jose, CA, USA; cat.# 610822); mouse monoclonal anti-lysosomal membrane glycoprotein (LAMP-1), clone ID4B (1:100; Developmental Studies Hybridoma Bank, Iowa City, IA, USA; cat.# ID4B); mouse monoclonal anti-KDEL (Lys-Asp-Glu-Leu motif) receptor (KDELR), clone KR-10 (1:50; Stressgen Biotechnologies, San Diego, CA, USA; cat.# VAA-PT048); mouse monoclonal anti-protein disulfide-isomerase (PDI), clone 1D3 (1:200; Enzo Life Sciences, Farmingdale, NY, USA; cat.# ADI-SPA-891); and mouse monoclonal anti-calcitonin gene-related peptide (CGRP; 1:100; Abcam, Cambridge, UK; cat.# ab81887).

Once washed with PBS, the sections were incubated for 1 h with a combination of appropriate secondary fluorescent antibodies labeled with one of the following fluorochromes (1:500): Alexa Fluor 488, Alexa Fluor 546 (Molecular Probes, Eugene, OR, USA), Cy3, or Cy5 (Jackson Immuno Research Laboratories, West Grove, PA, USA). In some experiments, DAPI (1:100; Sigma-Aldrich, Saint Louis, MO, USA) was also added to stain the cell nuclei. Finally, the spinal cord sections were counterstained with blue fluorescent NeuroTrace Nissl stain (1:150; Molecular Probes) and mounted using an anti-fading medium containing 0.1 M Tris-HCl buffer (pH 8.5), 20% glycerol, 10% Mowiol, and 0.1% 1,4-diazabicyclo[2, 2, 2]octane. Immunocytochemical controls were achieved by the omission of primary antibodies in parallel-processed sections resulting in no detectable staining.

The slides were examined under a FluoView FV-500 or FluoView FV-1,000 Olympus laser scanning confocal microscope (Olympus, Hamburg, Germany). For comparisons, slides from different animals and experimental conditions were processed in parallel for immunocytochemistry and subsequent imaging. The same scanning parameters were used for the acquisition of images corresponding to the different experimental groups. Z-stack confocal images of MN cell bodies (0.5–1-μm thick optical sections) were obtained. When required, Z-stacks are displayed as maximum intensity projections.

Brain and brainstem sections that were incubated with the Y172 antibody (1:1,000), were processed for immunoperoxidase staining according to standard ABC procedures (Vectastain, Vector, Burlingame, CA, USA). The sections were finally mounted on gelatin-coated slides, air-dried, and coverslipped with DPX. The sections were identified and mapped as described in the Allen Reference Atlas (Dong, 2008).

### Image and Morphometric Analysis

Digital images of the entire lumbar region were obtained from every 30th section and analyzed with ImageJ software (US National Institutes of Health, Bethesda, MD, USA). Only fluorescent Nissl stained MNs exhibiting a large nucleus, visible nucleolus, and large soma were analyzed. The area and perimeter of MN somata were manually measured. The number, area and fluorescent intensity of Y172-positive profiles, and of profiles expressing the different proteins examined and the degree of spatial association between them, were evaluated in binarized images obtained from multiple immunolabeled sections. Binarized Y172-positive profiles were used as a mask for measuring the immunostaining values of other proteins after splitting multichannel images. For immunofluorescence colocalization analysis, a plugin developed for ImageJ by Pierre Bourdoncle (bourdoncle@ijm.jussieu.fr) was used. In some cases, three-dimensional reconstructions were performed using Bitplane (Imaris, Bitplane, CT, USA) on a 0.5-μm-thick Z step obtained with a confocal microscope. The digital images were edited using FV10-ASW 3.1 Viewer (Olympus) and Adobe Photoshop CS4 (Adobe Systems Inc., San Jose, CA, USA). Although the investigators were not blinded to the experimental condition, to minimize bias, the MN selection for analysis was randomly performed in the fluorescent Nissl channel. In this way, observers were unaware of the pattern of immunostaining to be analyzed in the complementary channels. Experiments were examined by two independent investigators obtaining similar results.

### Electron Microscopy

Some mice were perfused with either 1% PFA and 1% glutaraldehyde in 0.1 M PB (pH 7.4) for conventional electron microscopy or with 4% PFA and 0.2% glutaraldehyde in 0.1 M PB (pH 7.4) for immunoelectron microscopy.

For conventional electron microscopy, dissected tissues were postfixed in 1% OsO_4_ and processed for Embed 812 embedding (Electron Microscopy Sciences, Fort Washington, PA, USA) according to standard procedures. Ultrathin sections were counterstained with uranyl acetate and lead citrate.

For ultrastructural Y172 immunolabeling, pre-embedding or post-embedding [freeze-substitution and low temperature embedding in Lowicryl HM20 resin (Electron Microscopy Sciences, Fort Washington, PA, USA)] procedures were used, as previously described (Gallart-Palau et al., 2014). To facilitate the localization of C-boutons in ultrathin cryosections, hypoglossal MNs rather than spinal cord MNs were examined in some cases. For this, brainstem or spinal cord samples were sectioned (50-μm thick for pre-embedding and 200 μm thick for post-embedding) with a vibratome, and the regions containing either the hypoglossal nucleus or the spinal cord ventral horn were microdissected.

Briefly, for pre-embedding immunolabeling, vibratome sections were treated with 50 mM glycine in PBS for 30 min, cryoprotected in 30% sucrose in 0.1 M PB and permeabilized with four freeze/thaw cycles in liquid N_2_. The sections were incubated overnight at 4°C with the Y172 antibody (1:50; Abcam or Millipore) and then for 1 h with an anti-rabbit biotinylated antibody (1:100; Vector), visualized using Vectastain Elite ABC (Vector), and incubated in a 0.05% 3,3-diaminobenzidine /0.01% H_2_O_2_ mixture. Peroxidase reactive sites were further subjected to silver enhancement to allow visualization by electron microscopy. Briefly, sections were incubated in a solution containing 2.6% hexamethylenetetramine (Merck, Darmstadt, Germany), 0.2% silver nitrate (Merck, Darmstadt, Germany) and 0.2% disodium tetraborate (Merck, Darmstadt, Germany) for 10 min at 60°C. The sections were then rinsed in distilled water and treated with 0.05% gold chloride (Merck) in distilled water for 2 min. Finally, the sections were rinsed, incubated in 2.5% sodium thiosulfate (Merck, Darmstadt, Germany) for 2 min, postfixed with 2% OsO_4_ in cacodylate buffer for 1 h, and dehydrated in a graded series of acetone. Labeled sections were flat embedded in Embed 812, sectioned and counterstained with uranyl acetate and lead citrate.

For post-embedding, ultrathin cryosections obtained with a Leica EM FC cryo ultramicrotome (Leica Microsystems, Wetzlar, Germany) were processed according to established procedures (Tokuyasu, 1997; Slot and Geuze, 2007) and double immunolabeled using the Y172 primary antibody (1:20; Abcam or Millipore) and a mouse monoclonal anti-SR1 (1:10; Santa Cruz Biotechnology, Dallas, TX, USA) primary antibody. After washing, the sections were incubated with 12-nm gold-conjugated goat anti-rabbit IgG (1:30; Sigma-Aldrich, Saint Louis, MO, USA) and/or 18-nm gold-conjugated goat anti-mouse IgG (1:30; Sigma-Aldrich, Saint Louis, MO, USA). Controls were achieved by omitting the primary antibody.

Observations were performed with a Jeol JEM 1400 (Jeol, Tokyo, Japan) transmission electron microscope.

### Western Blotting

Frozen spinal cords were fragmented and homogenized in blending buffer 1× SR (2% SDS and 125 mM Tris-HCl, pH 6.8) supplemented with protease inhibitor (Sigma, cat.# P8340) and PhosSTOP (Roche, Laval, QC, Canada). The homogenized samples were then heated to 100°C for 5 min and centrifuged at 12,000 rpm for 5 min. The protein concentrations of the supernatants were determined by BIO-RAD Micro DC protein assay (BIO-RAD, Laboratories Inc., Hercules, CA, USA). Loading buffer 4× SS (20% sucrose, 0.05% bromophenol blue, and 0.1% sodium azide) containing 5–10% β-mercaptoethanol (Sigma-Aldrich, Saint Louis, MO, USA) and 15 μg of protein was then loaded onto a 10% or 15% polyacrylamide electrophoresis gel. The proteins were electrotransferred to PVDF (ImmobilonTM-P, Millipore, Burlington, MA, USA) membranes in a Tris-glycine-methanol-buffered solution. The membranes were then blocked with 5% dried skimmed milk in 0.1% Tween 20 and Tris-buffered saline (TBST), pH 8, for 1 h at RT and then extensively washed in TBST. Immunodetection was performed by incubating the membranes overnight at 4°C with the following antibodies: Y172 (1:1,000, Abcam or Millipore), rabbit polyclonal anti-p-c-Jun (Ser63; 1:1,000; Cell Signaling, Danvers, MA, USA; cat.# 9261); rabbit polyclonal anti-p-c-Jun (ser 73; 1:1,000; Cell Signaling, Cell Signaling, Danvers, MA, USA; cat.# 9164); rabbit monoclonal anti-c-Jun (1:1,000; Cell Signaling, Danvers, MA, USA; cat.# 9165); and a mouse monoclonal anti-β-actin, which was used as a loading control. The membranes were washed in TBST, incubated for 60 min at RT with the appropriate peroxidase-conjugated secondary antibodies (1:5,000; Amersham Biosciences, Buckinghamshire, UK), washed in TBST, and visualized using the ECL Prime Western Blotting Detection Reagent Detection Kit (GE Healthcare, Buckinghamshire, UK) following the procedure described by the manufacturer. The quantification of band densities was performed by a using Chemi-Doc MP Imaging System (BIO-RAD Laboratories Inc., Hercules, CA, USA).

### Statistical Analysis

The data are expressed as the means ± standard error of the mean (SEM). Statistical analyses were performed by student’s *t*-test or either one-way or two-way analysis of variance (ANOVA) followed by *post hoc* Bonferroni’s test. The level of significance was established at *p* ≤ 0.05. GraphPad Prism 6 software was used for statistical analysis and the graphical presentation of the data.

## Results

### The Y172 Monoclonal Antibody Against p-c-Jun Unexpectedly Immunolabels C-Bouton Postsynaptic Sites in Spinal Cord MNs

**Figure 1:**
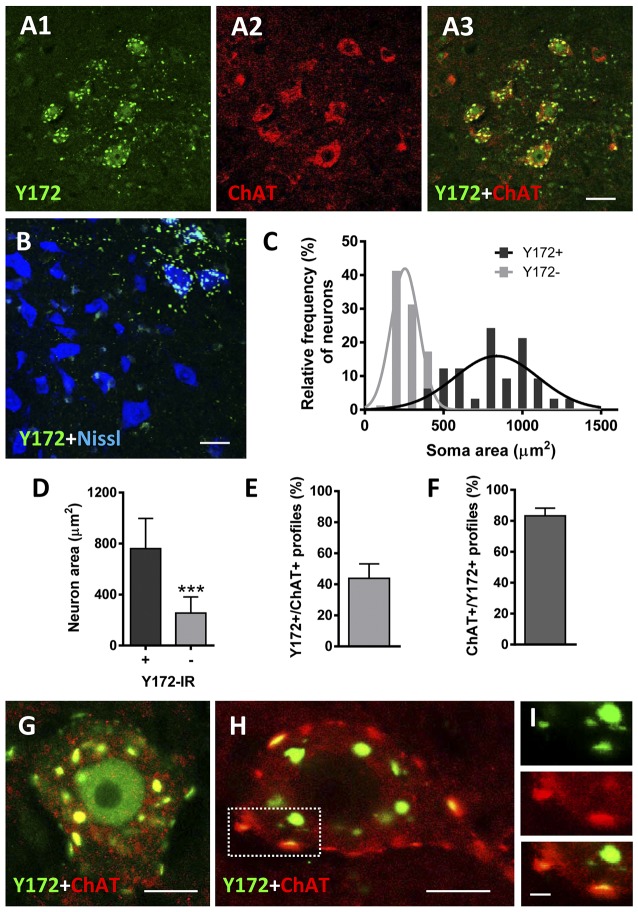
p-c-Jun-like immunoreactivity of the Y172 antibody in spinal cord MNs from adult CD1 mice (P75). **(A1–A3)** Representative confocal images are taken from cryostat sections double immunostained with Y172 (green) and anti-ChAT (red) antibodies. Note the Y172 immunolabeling in large-sized neurons in the ventral horn, which was immunostained for ChAT as an MN marker; Y172 immunoreactivity was also seen in the ventral horn gray matter around MNs. **(B)** Confocal micrograph obtained from a section immunolabeled with the Y172 antibody (green) and counterstained with fluorescent Nissl stain (blue) for neuron visualization. Note the selective distribution of Y172-positive immunolabeling in large-sized neurons with an angular-shaped cell body characteristic of MNs; smaller neurons, presumably γ-MNs or ventral interneurons, were devoid of Y172 immunostaining. **(C)** The soma size distribution of neurons showing either positive or negative immunostaining with the Y172 antibody; 200 MNs from three different animals were analyzed. Y172-positive and -negative neurons were fit to Gaussian curves representing large and small populations, respectively. **(D)** The average soma size of neurons exhibiting either positive or negative immunoreactivity with the Y172 antibody obtained from the data shown in **(C)**; ***p < 0.001 vs. Y172+ immunoreactivity (IR), student’s *t*-test. **(E,F)** The percentage of total Y172 positive profiles in the cytoplasm of MNs showing a spatial association with ChAT-positive profiles **(E)**, and ChAT-positive spots also displaying Y172 immunoreactivity **(F)**; Three hundred and 200 profiles from 10 randomly selected MNs were analyzed in **(E)** and **(F)**, respectively. **(G–I)** Representative images of MNs double immunostained with Y172 (green) and anti-ChAT (red) antibodies. Note the pattern of Y172 immunoreactivity in the form of positive spots of different sizes distributed in the cytoplasm, with some immunolabeled profiles located in the periphery of the cell bodies; most of the Y172-positive profiles, particularly those located peripherally (i.e., the dotted-lined square), exhibited a spatial association with ChAT, as shown in the higher magnification image **(I)**. Y172 immunolabeling was also observed in the nucleus of a limited number of MNs **(G)**. The data in the graph are expressed as the mean ± SEM. Scale bars: **A3** (valid for **A1,A2**) **B** = 30 μm; **G,H** = 10 μm; **I** = 2.5 μm.

p-c-Jun immunostaining using a rabbit monoclonal anti-p-c-Jun (Ser63) antibody clone Y172 (hereafter referred to as the Y172 antibody) in combination with an antibody against ChAT (the enzyme that catalyzes the synthesis of acetylcholine) as a MN marker and fluorescent Nissl staining for neuron visualization, was first analyzed in the lumbar spinal cord of adult (P75) CD1 mice by confocal microscopy. Y172 immunolabeling was observed in the form of a bright punctate pattern predominantly distributed in the ventral horn gray matter and particularly in a subpopulation of neurons showing large and angular-shaped somata by Nissl staining (Figures 1A1–B). These Y172-positive cells, which correspond to MNs based on their morphology and location, had a mean cross-sectional area of 760.77 μm^2^ (± 40.52 [SEM]) and exhibited strong ChAT immunoreactivity (Figures 1A1–D); all these features fit well with the previously reported defining criteria of α-MNs (Shneider et al., 2009). In contrast, smaller neurons (mean cross-sectional area of 254.25 ± 13.1 μm^2^) exhibited Y172-negative immunostaining (Figures 1B–D) and were considered either γ-MNs (ChAT-positive) or ventral interneurons (ChAT-negative; Shneider et al., 2009). In large MNs, Y172-positive immunolabeling was observed in granular structures of different sizes widely distributed in the cytoplasm and in the form of conspicuous puncta located in the periphery of the cell body (Figures 1G,H). Y172 immunopositivity was found in the nucleus of a small number (~9%) MNs (Figure 1G); this is in agreement with the findings of previous reports (Yuan et al., 2010), in which no p-c-Jun-positive nuclei were detected in spinal MNs from adult rats. In contrast, more than 95% of MNs displayed prominent cytoplasmic p-c-Jun-like immunoreactivity upon application of the Y172 antibody (Figures 1G,H). Image analysis showed that ~38% of Y172-positive spots in MN somata also displayed strong immunoreactivity for ChAT; moreover, the vast majority of ChAT-positive spots (~85%) exhibited Y172 immunoreactivity as well (Figures 1E,F,H,I).

**Figure 2:**
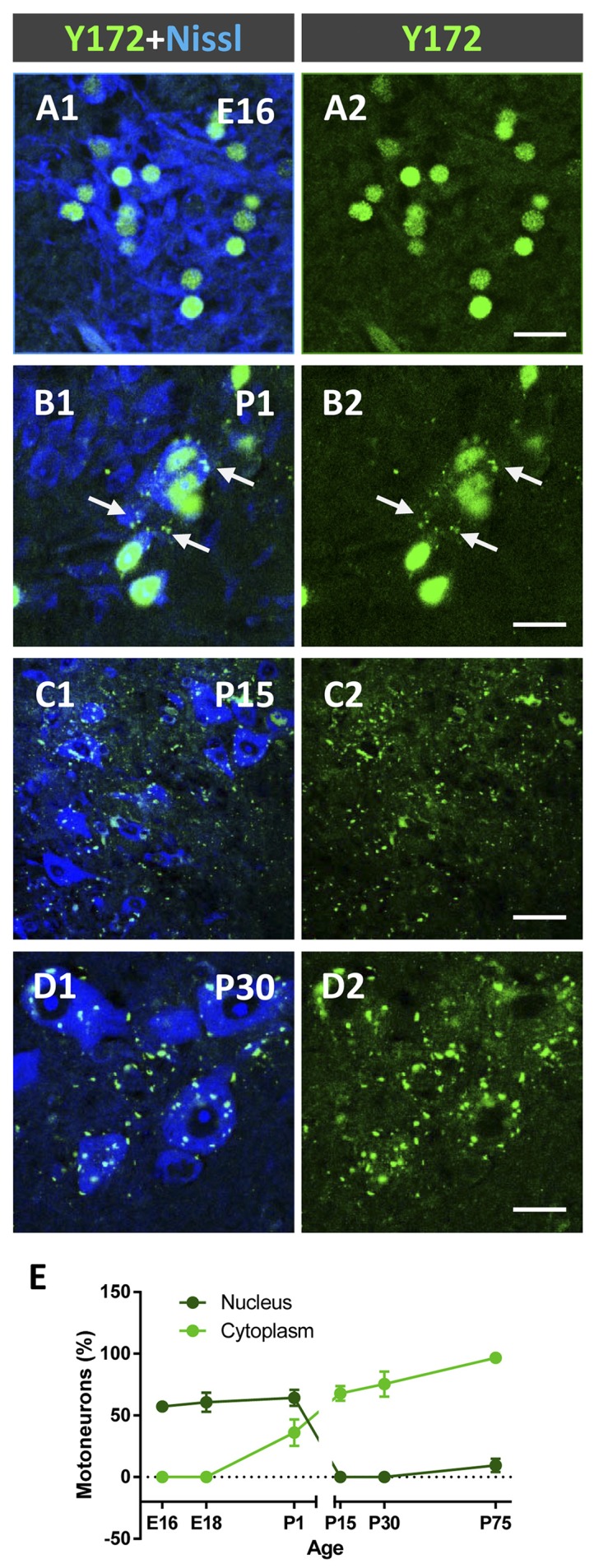
Developmental changes in p-c-Jun-like-immunoreactivity with the Y172 antibody in spinal cord MNs from E16-P30 CD1 mice. **(A1–D2)** Representative confocal micrographs were taken from cryostat sections immunolabeled with the monoclonal antibody (green) and counterstained with fluorescent Nissl stain (blue) for MN visualization. Note the selective nuclear location of Y172-positive immunostaining in MNs at E16 **(A1,A2)**; at P1, MN cell bodies exhibited small cytoplasmic Y172-positive spots (arrows), which coexisted with nuclear immunolabeling **(B1,B2)**. At P15 and P30, the vast majority of MNs did not exhibit nuclear immunostaining but showed abundant Y172-positive patches widely distributed in the cell body cytoplasm and in the ventral horn gray matter around MNs **(C1–D2)**. **(E)** The time course of developmental changes in Y172 immunoreactivity in MNs. The data are expressed as the mean ± SEM; 8–14 randomly selected sections per spinal cord from 3-4 different animals per age were analyzed. Scale bars: **A2,B2,D2** = 20 μm; **C2** = 40 μm.

Due to the striking and unexpected pattern of immunostaining obtained with the Y172 antibody, which had not been previously described for other antibodies against p-c-Jun, we decided to examine the changes in this immunostaining during MN development in CD1 mice (Figures 2A1–E). On E16 and E18, Y172-positive immunolabeling was observed in the nuclei of ~60% of MNs, but there was no detectable positivity in the cytoplasm of the cell bodies (Figures 2A1,A2). On P1, although the number of MNs with nuclear Y172 immunostaining was similar to that observed on E18, immunolabeled cytoplasmic spots were also detected in more than 35% of MNs, and some small positive puncta were found to be distributed in the ventral horn gray matter (Figures 2B1,B2). During the postnatal period, a drastic decrease in Y172 positivity was found in the nuclei of MNs; thus, from P15 on, almost none of these cells exhibited nuclear Y172 immunostaining (Figures 2C1,C2). The loss of nuclear Y172 immunoreactivity correlated with a gradual increase in immunoreactivity in the cytoplasm of MNs and in the ventral horn gray matter; thus, on P30, the pattern of p-c-Jun-like expression detected in MNs with the Y172 antibody was similar to that observed in the adult (P75) mouse spinal cord (Figures 2D1,D2, and compare with Figures 1A1–B). The time course of these changes is shown in Figure 2E.

We next explored whether a similar pattern of immunoreactivity can be observed with the same antibody in other animal species, such as rats and chickens. In both species, the Y172 antibody exhibited a comparable pattern of immunostaining and distribution in MNs and spinal cord gray matter as that seen in adult mice (Supplementary Figures S1A1–D2).

**Figure 3:**
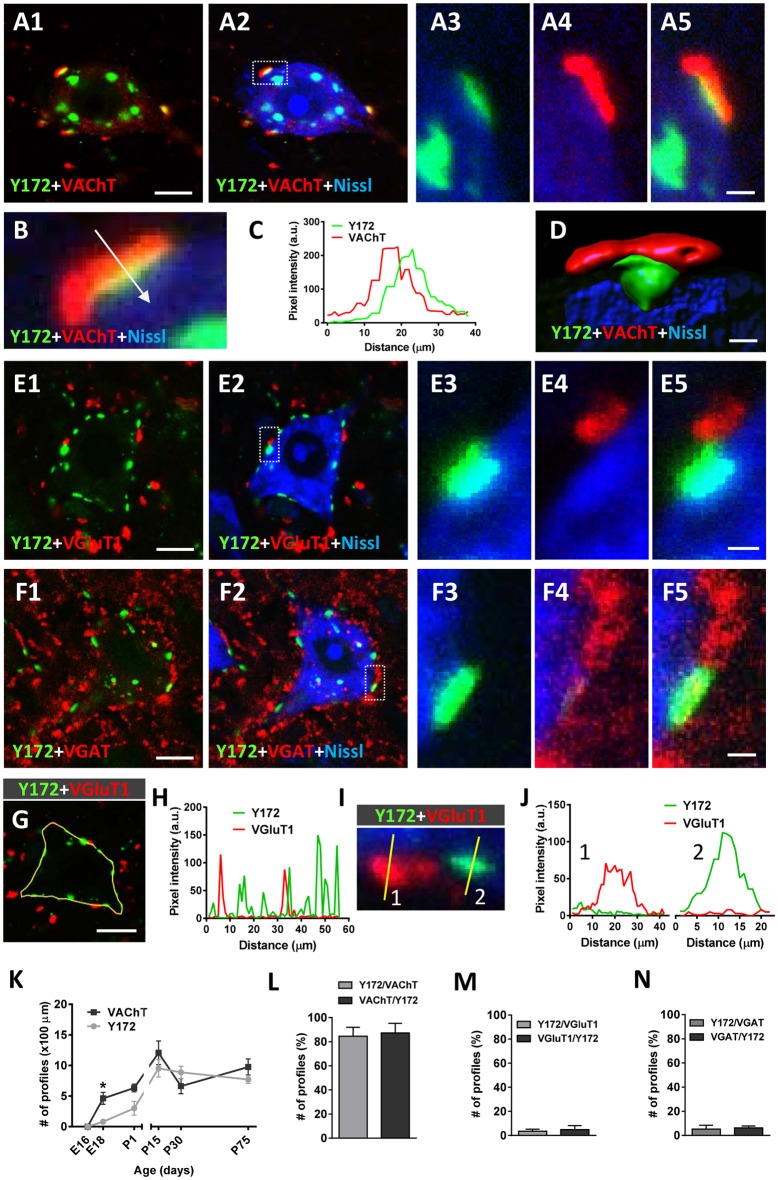
Immunocytochemical analysis of the spatial relationships between peripheral cytoplasmic Y172 immunolabeling and excitatory and inhibitory afferent inputs to MNs of CD1 mice. Spinal cord sections were double immunostained with Y172 (green) and either anti-VAChT, VGluT1 or VGAT (for cholinergic, glutamatergic or GABAergic synapses, respectively, all red) and processed for fluorescent Nissl staining for MN visualization (blue). **(A1–A5)** Representative maximum intensity projections from confocal Z-stacked images showing Y172 and VAChT immunoreactivity in an MN of an adult (P75) mouse. Note the distribution of Y172-positive spots in the cytoplasm of the cell body; while some immunoreactive patches were located around the nucleus, others were peripherally distributed and exhibited a close association with VAChT-positive puncta. The area delimited by the dotted-lined rectangle in **(A2)** is shown at higher magnification in **(A3–A5)**; note that, while the peripherally located Y172-positive spot was in contact with a VAChT-positive punctum, the spot that was more internally located did not exhibit any association with VAChT immunolabeling. **(B,C)** Pixel profile analysis **(C)** along a line crossing a multifluorescent-labeled VAChT/Y172 synapse (shown in **B**) demonstrating the dissociation of presynaptic VAChT immunostaining and postsynaptic Y172-positive staining; the blue channel, corresponding to fluorescent Nissl staining, is not included in the graph. **(D)** Volume rendering of a high magnification confocal image of a C-bouton double immunolabeled with anti-VAChT (red) and Y172 antibodies (green) demonstrating the nonoverlapping and separate distribution of both signals; the blue channel corresponds to fluorescent Nissl stain for MN visualization. **(E1–F5)** Representative Z-staked images showing MNs immunostained with Y172 and either anti-VGluT1 **(E1–E5)** or anti-VGAT **(F1–F5)** antibodies. Note that neither VGluT1- nor VGAT-containing puncta were associated with Y172-positive profiles; the occasional degree of pixel overlapping observed in some cases was due to the random close proximity between Y172 and VGluT1 or VGAT immunoreactivity. **(G–J)** Pixel profile analysis **(H,J)** along the lines depicted in **(G,I)**; in **(G)**, the yellow line delimits the periphery of an MN by passing through different Y172- and VGluT1-positive spots, whereas in **(I)**, the lines cross two spots with VGluT1 immunoreactivity (1, red) and one spot with Y172 immunoreactivity (2, green); note the absence of colocalization between the two signals in **(H,J)**. **(K)** The time course of changes in the number of Y172- and VAChT-positive profiles per 100 μm of soma perimeter in spinal cord MNs from mice at different ages. **(L–N)** The percentage of peripheral Y172-positive profiles closely associated with puncta positive for VAChT, VGluT1, or VGAT, and vice versa, in adult (P75) MNs is shown in **(L–N)**, respectively. The data are expressed as the mean ± SEM; 200–300 profiles from 10 to 15 randomly selected MNs (3–4 animals) per condition were analyzed; *p < 0.05 vs. Y172+ immunoreactivity; student’s *t*-test. Scale bars: **A1** = 10 μm (valid for **A2**); **D** = 1 μm; **E1,F1** = 10 μm (valid for **E2,F2**); **F5** = 1.5 μm (valid for **A3–A5,E3–E5,F3,F4**); **G** = 10 μm.

Since the peripheral punctate pattern of p-c-Jun-like immunolabeling in the cytoplasm of adult MNs obtained with the Y172 antibody displayed clear similarities to the distribution of afferent inputs on MN somata and proximal dendrites, we performed double immunofluorescence analyses by combining the Y172 antibody with an antibody against synaptophysin (a marker of afferent synapses on MN somata and proximal dendrites, Supplementary Figures S2A1–C). Following confocal imaging and quantitative analysis, we found that virtually all (99.13 ± 0.5, *n* = 31 MNs) Y172-positive profiles peripherally located in MN somata were in close contact with synaptophysin-positive puncta. We then assessed whether these Y172-positive profiles were associated with a specific type of afferent inputs by immunocytochemistry by combining the monoclonal antibody with anti-VAChT, -VGluT1 and -VGAT antibodies (for cholinergic C-type [C-boutons], glutamatergic and GABAergic synapses, respectively; Figures 3A1–N). More than 60% of total Y172-positive patches distributed in MN somata showed a spatial relationship with VAChT-positive presynaptic terminals (C-boutons), and nearly all (>90%) of these VAChT-positive presynaptic terminals were associated with peripheral Y172-positive profiles (Figures 3A1–D,K,L); the ~40% of Y172-positive spots in MNs that were not associated with VAChT-positive signal mainly belonged to those more internally located in the cell body, far from its periphery. The percentages of peripheral Y172-positive profiles in MN somata associated with VAChT-positive puncta, and vice versa, are shown in Figure 3L. Colocalization and pixel profile analysis in 1-μm-thick optical sections indicated that the vast majority of peripheral Y172-positive spots were located in front of and in close contact with presynaptic VAChT-immunoreactive puncta, suggesting a potential function for the Y172 antigen in cholinergic synapses (Figures 3A1–C); however, both VAChT- and Y172-positive signals were not strictly overlapped, indicating a clear spatial dissociation between them. Following 3D reconstruction, we found that c-Jun-like-containing structures closely complemented VAChT-containing terminals, as expected for the postsynaptic location of the Y172 signal (Figure 3D). We then examined age-related changes in the number of peripheral Y172- and VAChT-positive puncta in spinal cord MNs (Figure 3K) and noticed that they showed a similar time course as that reported for other C-bouton-associated proteins (Milan et al., 2015; Casanovas et al., 2017; Salvany et al., 2019).

In contrast to the close relationship found between Y172 immunolabeling and C-boutons, only a minor proportion of total Y172-positive patches exhibited some association with VGluT1 or VGAT afferent terminals (~3.5% and ~5%, respectively; Figures 3E1–J,M,N); moreover, only ~3.5% of VGluT1-positive (Figure 3M) and ~6% of VGAT-positive (Figure 3N) puncta showed a certain degree of spatial relationship with Y172-positive spots. In all cases, VGluT1- and VGAT-immunoreactive terminals were distributed on the surface of MN somata next to Y172-positive spots but never in front of them, indicating that Y172-positive spots are exclusively associated with cholinergic boutons. The fact that a minority of Y172-immunostained puncta were very close to VGluT1- and VGAT-containing boutons may have resulted in some artifactual pixel overlapping after double immunolabeling. Indeed, the pixel profile values along the line traced on the spots exhibiting VGluT1 or VGAT immunoreactivity and those showing Y172 positivity revealed a clear dissociation between both signals (Figures 3G–J, for VGluT1, not shown for VGAT).

**Figure 4:**
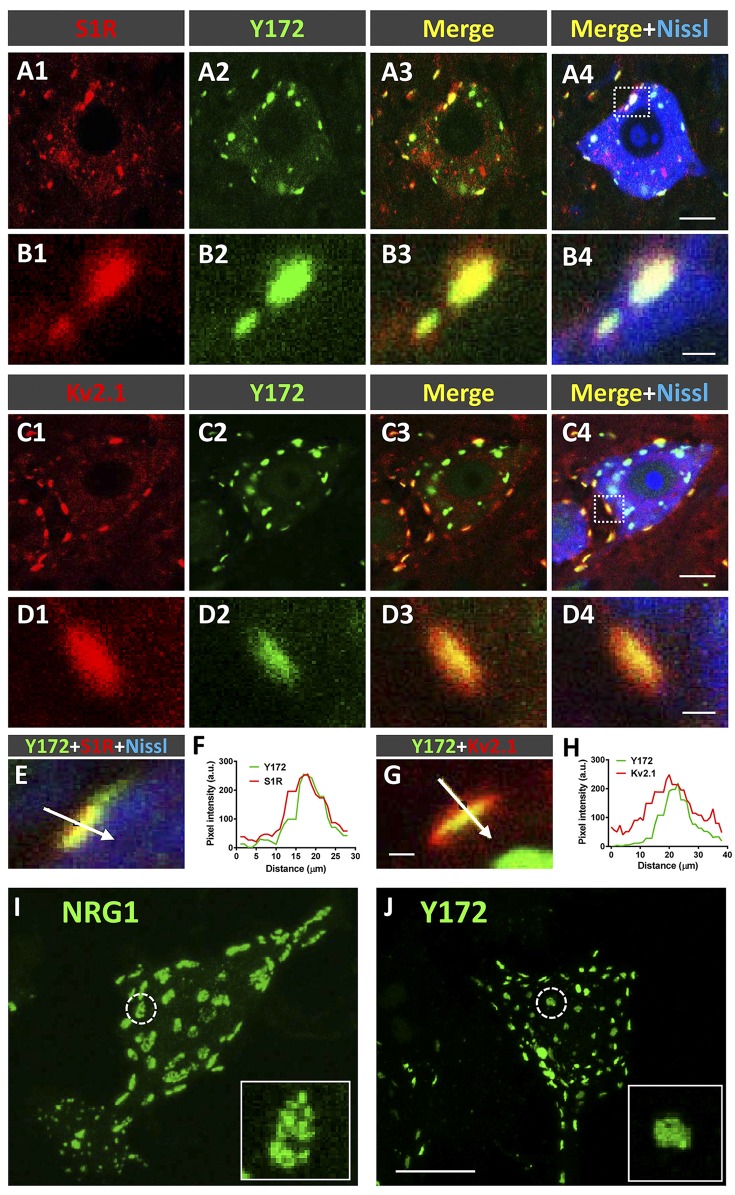
Y172 immunoreactivity colocalizes with S1R and Kv2.1 in C-boutons. **(A1–D4)** Representative confocal images of adult (P75) mouse spinal cord MNs double immunostained with antibodies against either S1R or Kv2.1 (red) and Y172 (green), as indicated in the panels; sections were counterstained with fluorescent Nissl stain (blue) for MN visualization. The areas delimited by dotted-line squares in **(A4,C4)** are shown at higher magnification in **(B1–B4,D1–D4)**, respectively. **(E–H)** Pixel profile analysis **(F,H**) along the lines depicted in **(E,G)**, which were obtained from MNs immunolabeled with Y172 (green) and anti-S1R or Kv2.1 (red) antibodies; fluorescent Nissl staining (blue) for MN visualization can be seen in **(E)**. Note the overlap between the Y172 and S1R **(F)** or Kv2.1 **(H)** signals. **(I,J)** Squashed MNs immunolabeled with anti-NRG1 **(I)** and Y172 **(J)** antibodies; the circled profiles are shown at higher magnification in the insets; note that Y172-positive profiles were smaller and displayed more compact patterns than NRG1 clusters. Scale bars: **A4,C4** = 10 μm (valid for **A1–A3,C1–C3**); **B4,D4** = 1 μm (valid for **B1–B3,D1–D3**); **G** = 1 μm (valid for **E**); and **J** = 20 μm (valid for **I**).

To further explore the relationship between Y172 immunolabeling and C-boutons in MNs, spinal cord sections from adult CD1 mice were incubated with the monoclonal antibody together with specific antibodies against S1R, Kv2.1 (two proteins known to be located at the postsynaptic compartment of C-boutons) and VAChT (Figures 4A1–H). We found that 65.55% (± 6.60, mean ± SEM) of S1R-immunostained spots and 86.39% (± 5.04) of Kv2.1-immunostained spots were associated with Y172-positive puncta (*n* = 224 S1R-containing puncta and 103 Kv2.1-containing puncta from 8 and 6 MN somata, respectively). Pixel profile analysis indicated an overlap between S1R and Kv2.1 signals and Y172 immunostaining (Figures 4E–H), indicating that all of them are distributed postsynaptically at C-boutons. We described that SR1 and Kv2.1 reside in C-bouton SSC in nonoverlapping microdomains in conjunction with NRG1 (Casanovas et al., 2017). As the anti-NRG1 antibody that works well for C-bouton labeling is hosted in rabbits, double immunolabeling with the Y172 and anti-NRG1 antibodies was not feasible. However, by analyzing individual MNs after single immunolabeling, we noticed that NRG1 and the Y172 antigen did not share the same microdomain at the C-bouton postsynaptic site. In fact, NRG1 clusters were significantly larger than Y172-positive spots (NRG1: 7.15 ± 0.78 μm^2^; Y172: 1.98 ± 0.05 μm^2^; *p* < 0.001; *n* = 11 puncta per condition in orthogonal projections) and displayed a reticular micropunctate pattern compared to the more compact and homogeneous appearance of Y172 puncta (Figures 4I,J).

We also explored whether specific labeling in spinal cord MNs with the Y172 antibody is also present in cranial MNs. We found that MNs from facial (CN VII), ambiguous (CNX), hypoglossus (CN XII), and trigeminal (CNV) nuclei displayed an identical punctate cytoplasmic pattern of Y172 immunoreactivity. This appeared to sometimes be associated with a weak nuclear Y172 signal, as expected for p-c-Jun. In contrast, MNs from nuclei that innervate extrinsic ocular muscles, such as the oculomotor (CN III), trochlear (CNIV), and abducens (CNVI) nuclei, exhibited higher nuclear Y172 positivity, but the punctate cytoplasmic pattern was virtually absent. This is consistent with the described absence of C-bouton afferents in ocular MNs (Hellström et al., 2003; Gallart-Palau et al., 2014). Neurons located in nonmotor areas occasionally displayed nuclear Y172 immunoreactivity without any clustered cytoplasmic signal (Supplementary Figure S3).

### Cellular Compartmentation of Y172 Immunolabeling in Spinal Cord MNs From CD1 Mice

**Figure 5:**
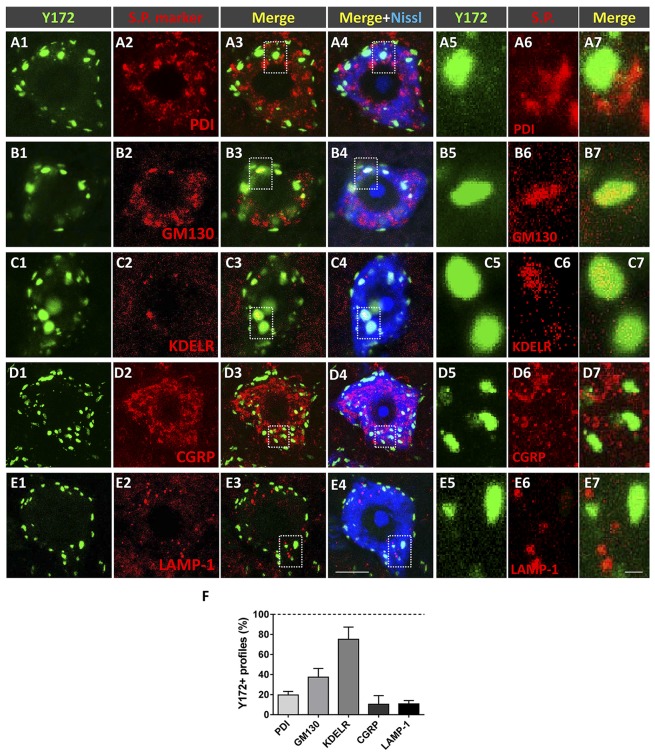
Nonsynaptically associated cytoplasmic Y172 immunoreactivity in MNs is mainly located in secretory pathways (s.p.). **(A1–E7)** Representative confocal micrographs of adult (P75) mouse MNs in spinal cord sections double immunostained with the Y172 antibody (green) and for different intercellular compartment markers (red) including PDI, GM130, KDELR, CGRP, and LAMP1 (as indicated in the panels); sections were counterstained with fluorescent Nissl stain (blue) for MN visualization. The areas delimited by dotted-line squares in **(A3–E4)** are shown at higher magnifications in **(A5–E7)**. **(F)** The percentage of Y172-positive puncta that colocalized with the different markers of secretory pathway compartments. The data are expressed as the mean ± SEM; 200–300 Y172-positive profiles in 10 randomly selected MNs from three mice were analyzed. Scale bars: **E4** = 10 μm (valid for **A1–E3**); **E7** = 1 μm (valid for **A5–E6**).

To examine the cellular location and significance of nonsynaptically associated Y172-immunoreactive cytoplasmic spots, we performed immunofluorescent analysis by using the monoclonal antibody in combination with specific antibodies against different secretory pathway compartments, including PDI (an ER chaperone responsible for the formation of disulfide bonds in proteins, Perri et al., 2016), GM130 (a *cis*-Golgi matrix protein that is involved in the maintenance of the structure of the Golgi apparatus and in the fusion of vesicles to its membrane, Nakamura, 2010), KDELR (a marker of the intermediate compartment of the Golgi, Yamamoto et al., 2003), CGRP (which accumulates in trans-Golgi stacks and secretory vesicles, Calderó et al., 1992; Tarabal et al., 2001), and LAMP-1 [a major constituent of the lysosomal membrane (Eskelinen et al., 2003); Figures 5A1–F]. As expected, all these antibodies displayed an immunocytochemical pattern of fluorescent patches of different sizes that were widely distributed in the cytoplasm of MN cell bodies. Image analysis revealed that a high percentage (~75%) of patches immunoreactive for the Golgi marker KDELR also exhibited Y172 positivity, whereas a lower proportion (~40%) of GM130-immunolabeled profiles were also immunostained with the Y172 antibody. The great majority of Y172-immunoreactive profiles usually displayed a larger contour than those showing a KDLER- or GM130-positive signal. Conversely, a smaller percentage of profiles expressing either CGRP, the ER marker PDI or the lysosomal marker LAMP-1 (approximately 10%, 20% and 10%, respectively) also exhibited some Y172 positivity (Figure 5F). These results suggest that the vast majority of Y172-positive profiles are linked to structures of the intermediate compartment of the Golgi containing KDELR, which has been reported to recognize KDELmotif-containing proteins and promote their retrograde transport from the Golgi complex to the ER (Lewis and Pelham, 1990, 1992), and to GM130, which is strongly attached to membranes of the *cis*-side of Golgi stacks (Nakamura, 2010).

### Ultrastructural Localization of Y172 Immunoreactivity in MNs

**Figure 6:**
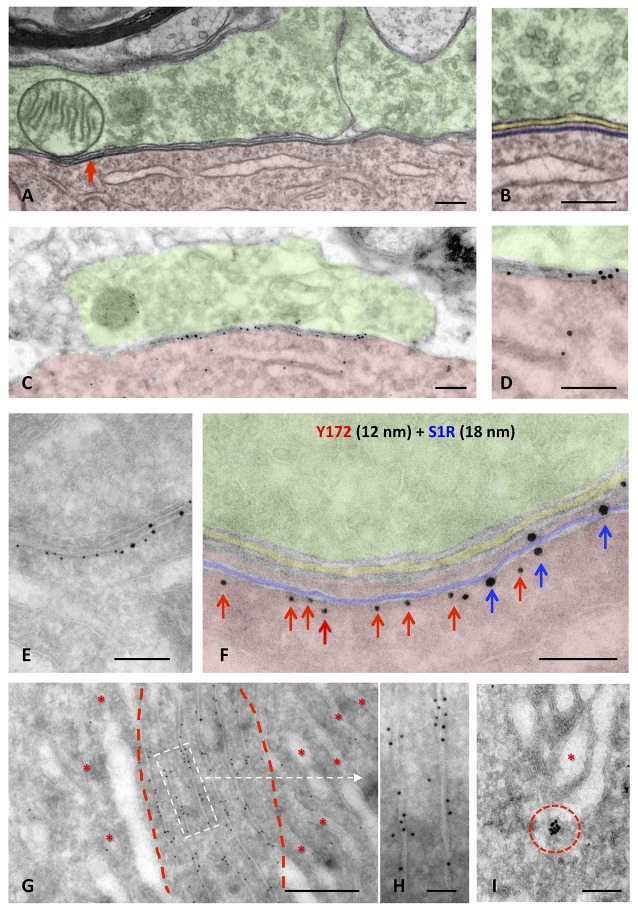
Ultrastructural localization of Y172 immunogold staining in the spinal cord and hypoglossal MNs of adult (P75) mice. **(A,B**) A C-bouton on a spinal cord MN cell body (shaded in red) observed by the electron microscopy after conventional processing; presynaptic terminals (shaded in green) were filled with small clear synaptic vesicles and some mitochondria. Adjacent and closely associated with the postsynaptic membrane, a flattened cistern of the endoplasmic reticulum (ER; shaded in blue), corresponding to the subsurface cistern (SSC), was observed (more detailed in **B**); the intersynaptic extracellular space is shaded in yellow. **(C,D**) A C-bouton detected in an ultrathin cryosection of an MN after double immunolabeling with different sizes of nanogold particles depicted at middle magnification. To facilitate the identification of C-bouton synaptic organization in a less structurally defined cryosection, compare **(C,D)** with **(A,B)**. Note also the accumulation of gold particles representing Y172 immunoreactivity at the postsynaptic region of the C-bouton (shaded in red). **(E,F)** A higher magnification image showing a double immunogold labeling of a C-bouton synaptic site using the Y172 antibody (red arrows) and an anti-S1R antibody as SSC marker (blue arrows), visualized with 12-nm and 18-nm particles, respectively. To facilitate the localization of the immunolabeling, the coloring of the synaptic subcompartments in **(F)** is the same as that in** (A,B)**; Y172 antibody immunoreactivity was located on the outer surface of SSC membrane (red arrows) and clustered separately from S1R immunoreactivity (blue arrows). **(G,H)** Y172 immunoreactivity was also associated with stacked membrane sheets of the ER (delimited by red dashed lines); other ER regions containing less compact cisterns (*) were devoid of labeling. **(H)** A detailed image of the ER (delimited by the white dashed-line rectangle in **G**) immunolabeled with the Y172 antibody revealed that gold particles were mainly associated with the external surface of the ER membranes. **(I)** Y172 immunolabeling was also observed in vesicles (delimited by the red dashed circle) adjacent to the Golgi apparatus (*). Scale bars: **A–F** = 200 nm; **G** = 500 nm; **H,I** = 100 nm.

To analyze the subcellular location of Y172 immunoreactivity in MNs, ultrastructural analysis of the adult spinal cord and hypoglossal MNs was performed. Hypoglossal MNs display C-boutons that are more densely packed than and essentially identical to those located in spinal cord MNs (Gallart-Palau et al., 2014), facilitating C-bouton sampling in ultrathin sections. The ultrastructural appearance of a C-bouton contacting a spinal cord MN is shown in Figures 6A,B; as previously widely described, C-bouton presynaptic terminals appeared to be filled with small clear synaptic vesicles, whereas opposing postsynaptic counterparts exhibited the corresponding SSC, located very close to the MN membrane. High-resolution microscopy was performed using pre-embedding (immunoperoxidase) and post-embedding (immunogold staining on ultrathin cryosections) procedures. In concordance with the results obtained by light microscopy, ultrastructural immunolabeling showed an exquisite Y172 positivity associated with the postsynaptic region of C-boutons and to some intracellular membrane compartments in MN somata (Supplementary Figure S2). The highest resolution results were obtained by the post-embedding cryo-immunogold procedure. In this case, Y172-positive sites were clearly located at the cytoplasmic surface of SSCs (Figures 6C,D). By double immunolabeling for Y172 and the SSC marker S1R, it was observed that SSC microdomains enriched in the Y172 antigen were segregated from those in which S1R was concentrated (Figures 6E,F). This indicates that the Y172 antigen shares the mosaic-like distribution of other SSC-specific proteins (Deardorff et al., 2013, 2014; Casanovas et al., 2017). In addition to being found in synapses, Y172 positivity was also detected in particular ER regions in the cytoplasm of MN cell bodies corresponding to areas in which ER membranes displayed highly stacked motifs. In contrast, ER regions showing more dispersed cisterns were devoid of Y172 immunolabeling (Figure 6G). Gold particles were found predominantly on the cytoplasmic face of ER stacks (Figure 6H). Y172-immunoreactive sites were also observed to be associated with some Golgi profiles and were predominantly concentrated in some Golgi-associated vesicles (Figure 6I).

### Changes in Y172 Immunoreactivity in MNs After Peripheral Nerve Injury

**Figure 7:**
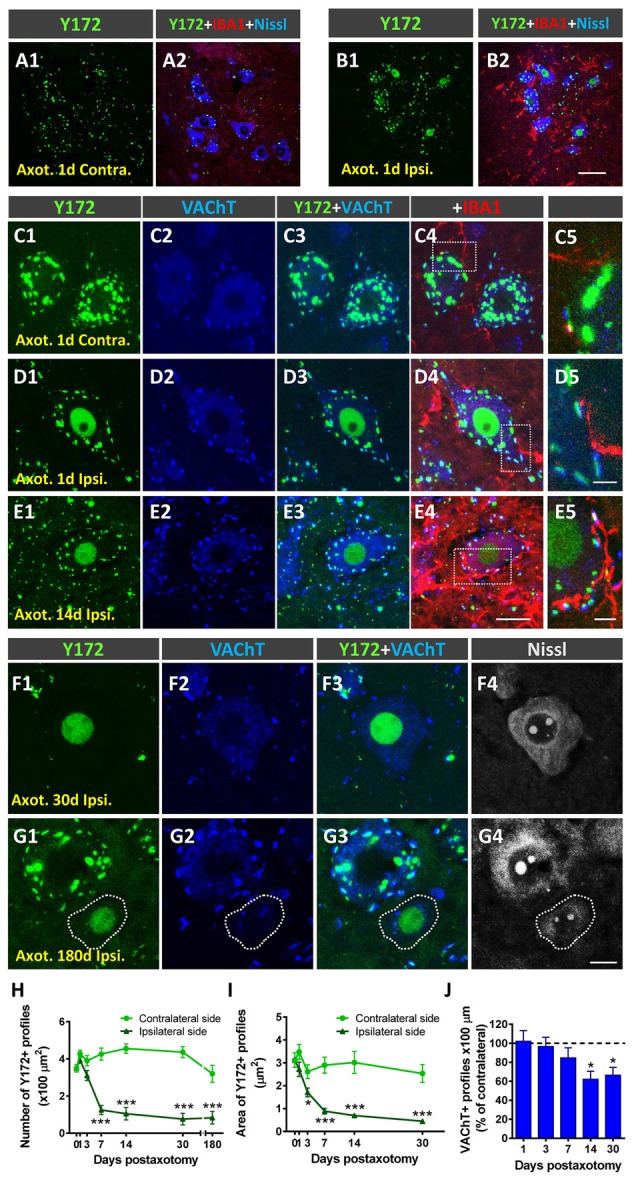
Changes in Y172 immunoreactivity in axotomized MNs. The sections of the spinal cord from adult (P60) mice subjected to unilateral sciatic nerve transection were immunostained with the Y172 antibody in combination with antibodies against VAChT (blue) and IBA1 (red), as indicated in the panels, to identify C-boutons and microglia, respectively; sections were also counterstained with fluorescent Nissl stain for MN visualization (shown in gray in **F4,G4**). **(A1–B2)** Ventral horn MNs in general view **(A1–B2**) and higher magnification **(C1–E5)** images from the contralateral (contra., nonaxotomized, **A1, A2,C1–C5**) and ipsilateral (ipsi., axotomized, **B1, B2,D1–E5**) sides of the spinal cord 1 **(A1–D5)** and 14 **(E1–E5)** days after nerve transection; note the nuclear Y172 immunostaining **(B1,D1,E1)** and the prominent IBA1-positive reactive microglia on the ipsilateral side **(B2, D4,E4)** in relation to those on the contralateral side **(A1,A2,C1,C4)**; in **(E1)**, an MN with intense nuclear Y172 staining as well as numerous profiles immunoreactive with the monoclonal antibody can be seen. The areas delimited by dotted-line rectangles in **(C4,D4,E4)** are shown at higher magnifications in **(C5,D5,E5)**; note, on the ipsilateral side **(D4,E4)**, the presence of abundant IBA1-positive microglial processes enwrapping MNs and contacting Y172-positive profiles, suggesting a role for microglia in the loss of peripheral cytoplasmic Y172 immunolabeling in axotomized MNs. **(F1–G4)** MNs on the ipsilateral side of spinal cord 30 **(F1–F4)** and 180 **(G1–G4)** days after axotomy; note in **(F1–F4)** the intense nuclear signal and the depletion of cytoplasmic peripheral profiles positive with the Y172 antibody. In **(G1–G4)**, an apparently healthy MN exhibiting both abundant cytoplasmic profiles and the absence of nuclear immunostaining with the Y172 antibody can be seen in the vicinity of an atrophic MN showing intense nuclear Y172 immunoreactivity. In all cases, MNs with nuclear Y172 signal displayed a reduced density of VAChT-positive C-boutons. **(H,I**) The density (per 100 μm^2^ of cell body) and size (in μm^2^) of cytoplasmic Y172-positive profiles in MNs located on the contralateral and ipsilateral sides of the spinal cord on different days after unilateral sciatic nerve transection. **(J)** Changes (expressed as the % of the contralateral side) in the density of VAChT-positive puncta observed in ipsilateral side MNs on different days post-axotomy. The data in the graph are expressed as the mean ± SEM, *p < 0.05 and ***p < 0.001 vs. the contralateral side; *n* = 8–11 randomly selected MNs per side from three mice per day post-axotomy. Scale bars: **B2** = 40 μm (valid for **A1–B1**); **E4** = 20 μm (valid for **C1–C4,D1–D4,E1–E3**); **E5** = 5 μm (valid for **C5,D5,E5**); **G4** =10 μm (valid for **F1–G3**).

Peripheral nerve interruption results in noticeable structural and metabolic changes in axotomized MNs (Lieberman, 1971) that are accompanied by a perineuronal glial reaction (Blinzinger and Kreutzberg, 1968; Aldskogius and Kozlova, 1998), the loss of proprioceptive inputs (Alvarez et al., 2011; Rotterman et al., 2014), and increased neuronal excitability (Romer et al., 2014). We (Casanovas et al., 2017; Salvany et al., 2019) and others (Romer et al., 2014) have reported the disruption and loss of cholinergic C-boutons and changes in their associated molecules in axotomized MNs. Due to the close spatial association between Y172 immunoreactivity and C-boutons, we next studied whether the cholinergic deafferentation of MNs induced by peripheral nerve injury is accompanied by changes in the immunocytochemical pattern observed upon staining with the Y172 antibody. It is known that, in adult MNs, axotomy provokes the rapid upregulation of c-Jun (Jenkins and Hunt, 1991; Wu et al., 1994), which is assumed to be associated with nerve regeneration (Ruff et al., 2012). The phosphorylation of c-Jun at Ser-63 and/or Ser-73 enhances its transcriptional activity (Dérijard et al., 1994; Smeal et al., 1994), and it has been reported that root avulsion, but not distal axonal lesion, induces the phosphorylation of this transcription factor in adult rat MNs (Yuan et al., 2010). We performed unilateral sciatic nerve transection and analyzed p-c-Jun-like immunoreactivity with the Y172 antibody on different days following lesion; this antibody was combined with antibodies against VAChT and IBA1 to identify C-boutons and microglia, respectively (Figures 7A1–G4). Axotomized MNs can be recognized since they are enwrapped by abundant reactive microglia, which display apparent positive chemoattraction to C-boutons (Casanovas et al., 2017). Twenty-four hours after axotomy, the ipsilateral side of the spinal cord exhibited numerous MNs showing positive nuclear Y172 immunostaining and increased IBA1 immunolabeling indicative of reactive microgliosis (Figures 7A1–D5). In axotomized MNs, the appearance of nuclear Y172 positivity was accompanied by a rapid reduction (~65%, 7 days post-axotomy) in both the density and size of cytoplasmic Y172-immunostained profiles (Figures 7H,I) and an enhancement of the recruitment of activated microglial cells around MN somata. At later time-points (14–180 days), the Y172-positive signal persisted in the nucleus but was barely detectable in the cell body cytoplasm of the vast majority of axotomized MNs (Figures 7F1–F4). The lack of cytoplasmic Y172-positive profiles in long-term axotomized MNs (i.e., 30–180 days) differed from their abundance in apparently nonaxotomized MNs, which did not exhibit a nuclear Y172-positive signal (Figures 7G1–G4). The time course of changes in the density and size of Y172-positive spots after axotomy are shown in Figures 7H,I. Compared with the progressive loss of C-boutons in MNs following nerve injury (Figure 7J), the disappearance of cytoplasmic Y172-positive profiles occurred more abruptly; thus, axotomized MNs showing very scarce spots immunostained with the Y172 antibody (e.g., 30 days after lesion) still maintained numerous VAChT-positive puncta on the cell body (compare Figure 7H with 7J). This indicates a clear dissociation between changes in structures exhibiting a Y172-positive signal and cholinergic deafferentation that occurred in injured MNs. Nevertheless, 180 days post-axotomy, ~60% of scarce cytoplasmic spots showing Y172 positivity in some axotomized MNs still maintained a spatial association with VAChT-immunoreactive puncta (ipsilateral side: 58.1% ± 11.8; contralateral side: 68.3% ± 4.42; *n* = 32 and *n* = 176 Y172-positive profiles from 5 and 7 MNs, respectively; *p* > 0.05, student’s *t*-test).

### Y172 Immunoreactivity in MNs Overexpressing NRG1 Type III Isoform

**Figure 8:**
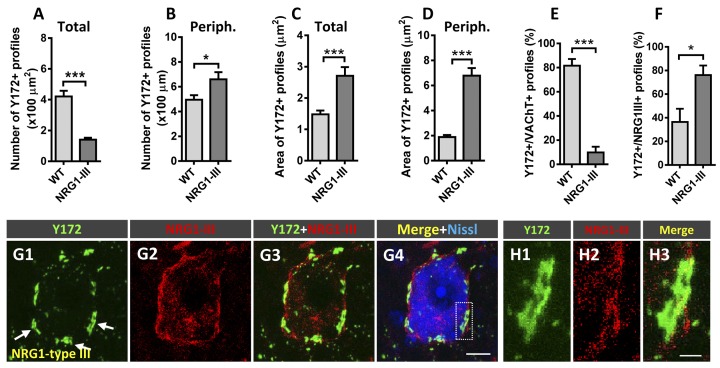
Changes in Y172 immunoreactivity in MNs from mutant mice (P60) overexpressing NRG1 type III. **(A–F)** The density (per 100 μm^2^ MN soma, **A,B**) and size (in μm^2^, **C,D**) of total **(A,C)** and peripheral (periph., **B,D**) Y172-positive profiles and the percentage of these profiles showing a spatial association with VAChT-positive C-boutons **(E)** and NRG1 type III-positive spots **(F)** in MNs from WT and NRG1 type III-overexpressing mice. Note that NRG1 type III overexpression was associated with a prominent decrease in the density of total Y172-positive profiles **(A)** and a significant increase in the number of those located peripherally **(B)** in MNs; the area of both total and peripheral Y172-positive profiles was dramatically increased in MNs from NRG1 type III-overexpressing animals **(C,D)**. Additionally, the percentage of Y172-positive profiles showing a close association with VAChT- **(E)** or NRG1 type III-positive **(F)** spots significantly increased or decreased, respectively, in MNs from NRG1 type III-overexpressing animals; 10–15 randomly selected MNs from 3 to 4 mice per condition were analyzed; *p < 0.05 and ***p < 0.001 vs. WT; student’s *t*-test). (**G1–G4)** Representative confocal micrographs of an NRG1 type III-overexpressing MN immunostained with Y172 (green) and anti-NRG1 type III (red) antibodies and counterstained with fluorescent Nissl stain (blue) for neuron visualization. Note that, compared to MNs of adult CD1 mice (see, for instance, Figure 1 or Figure 3), MNs overexpressing NRG1 type III exhibit an enlargement of Y172-positive profiles located in the periphery of the cell body, which correlated with the redundant and expanded SSCs previously described in Salvany et al. (2019); note also the expansion of NRG1 type III immunolabeling peripherally located in MN soma. **(H1–H3)** A higher magnification image of the area delimited in **(G4)** by the dotted-line rectangle corresponding to Y172, NRG1 type III and merged channels, as indicated, is shown. Scale bars: **G4** = 10 μm (valid for **G1-G3**); **H3** = 2.5 μm (valid for **H1, H2**).

NRG1 is a prominent component of C-boutons that specifically accumulates in SSCs of spinal MNs (Gallart-Palau et al., 2014; Casanovas et al., 2017). Studies performed by our group in transgenic mice overexpressing different NRG1 type isoforms have revealed that NRG1 type III acts as a specific organizer of SSC-like structures, whereas NRG1 type I promotes cholinergic synaptogenesis on MNs (Salvany et al., 2019). Here, we took advantage of mice overexpressing N-terminally HA epitope-tagged full-length NRG1 type III (NRG1-III; Velanac et al., 2012) to examine whether changes in NRG1 type III expression can influence Y172 immunoreactivity in MNs. We previously reported that NRG1 type III overexpression results in the accumulation of unprocessed NRG1 type III at the postsynaptic sites of C-boutons. This is accompanied by an expansion of surface-associated ER membranes in the form of redundant SSCs and the augmented expression of the SSC-associated molecules S1R and Kv2.1 and postsynaptic membrane-linked M2 muscarinic receptors; conversely, no changes in the density of cholinergic (VAChT-positive) C-boutons have been observed following NRG1 type III overexpression (Salvany et al., 2019). In the present experiments, we corroborated these findings (not shown) and detected significant changes in Y172 immunolabeling. Whereas the density of total cytoplasmic Y172-positive profiles was dramatically reduced in NRG1 type III-overexpressing MNs (Figure 8A), these MNs exhibited a marked increase in the density of peripheral Y172-positive spots compared to those exhibited by MNs from WT animals (Figure 8B). Moreover, Y172-positive profiles located in the periphery of NRG1 type III-overexpressing MN somata displayed a morphological pattern of enlargement (Figures 8C,D; see also Figures 8G1–H3), which correlated with the redundancy and expansion of SSC structures we previously described in this paradigm (Salvany et al., 2019). Colocalization analysis of the Y172 signal with either VAChT- or NRG1 type III-immunolabeling was performed in MNs from NRG1 type III-overexpressors and WT mice. We found that, in NRG1-type III-overexpressing MNs, the percentage of Y172-positive patches showing close contact VAChT-containing puncta was dramatically reduced (Figure 8E), whereas the proportion of Y172-positive patches associated with NRG1 type III-positive profiles was markedly increased (Figure 8F). These results indicate that an abnormal expansion of the SSC, such as that which occurs in NRG1 type III-overexpressing MNs, results in elevated Y172 immunoreactivity, presumably concentrated in its enlarged cisterns. Although closely related, NRG1 and the Y172 antigen appear to be clearly segregated in a mosaic-like form in SSCs.

### Y172 Immunoreactivity in Diseased MNs

We and others have reported that changes in C-boutons occur in murine models of the MN diseases ALS (SOD1^G93A^ mice) and SMA (SMNΔ7 and *Smn*^2B/-^ mice; Pullen and Athanasiou, 2009; Saxena et al., 2013; Gallart-Palau et al., 2014; Milan et al., 2015; Cerveró et al., 2018). Here, we wanted to examine whether similar changes in Y172 immunoreactivity also occur in these paradigms. Spinal cord sections from mutant SOD1^G93A^ and *Smn*^2B/-^ mice and their respective WT littermates were examined following double immunostaining with the Y172 antibody and an anti-VAChT antibody.

**Figure 9:**
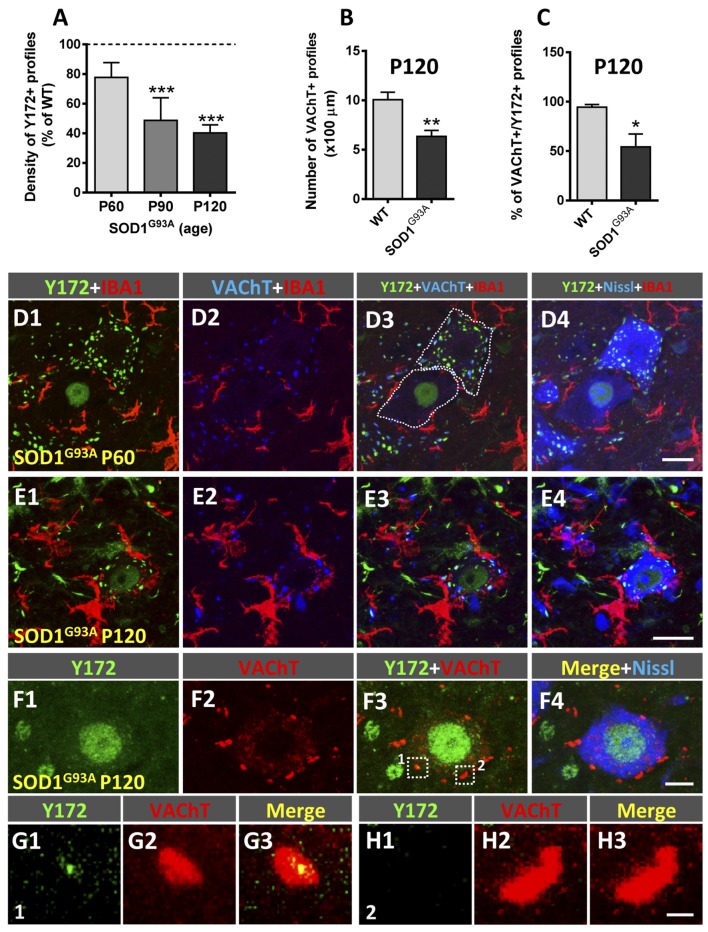
Changes in Y172 immunoreactivity in MNs of SOD1^G93A^ mice. **(A–C)** The density (number per 100 μm of soma perimeter) of peripheral Y172-positive profiles (shown as the % of WT, **A**) and of VAChT-positive puncta **(B)**, and the percentage of VAChT-positive puncta closely associated with Y172-positive profiles **(C)** in WT and mutant MNs at different stages of disease (presymptomatic [P60], early symptomatic [P90], and end-stage [P120]). The data are expressed as the mean ± SEM; 15–22 MNs randomly selected from SOD1^G93A^ animals and respective WT littermates were analyzed; *p < 0.05, **p < 0.01 and ***p < 0.001 vs. WT, student’s *t*-test. **(D1–E4)** Representative confocal micrographs of spinal cord sections from SOD1^G93A^ mice at the presymptomatic (P60, **D1–D4**) and end (P120, **E1–E4**) stages of disease immunostained with Y172 (green), anti-IBA1 (for microglia, red) and anti-VAChT (for C-boutons, blue) antibodies, as indicated in the panels; sections were also counterstained with fluorescent Nissl stain (blue in **D4**) for MN visualization. Note the presence of two MNs (delimited with dotted lines in **D3**), one of them exhibiting a normal appearance with abundant Y172-positive profiles widely distributed in the cell body cytoplasm and another with a typical appearance displaying intense nuclear labeling with the Y172 antibody and almost a complete absence of cytoplasmic profiles immunostained with this antibody, in **(D1–D4)**; there were numerous IBA1-positive microglial processes around the second type of MN, suggesting incipient degenerative changes. Prominent reactive microgliosis found around degenerating MNs exhibiting nuclear Y172 immunostaining in a P120 SOD1^G93A^ mouse is shown in **(E1–E4)**. **(F1–F4)** A spinal cord MN from a SOD1^G93A^ mouse at end-stage (P120) showing strong nuclear Y172 immunoreactivity (green) but depletion of cytoplasmic profiles immunostained with the Y172 antibody; this MN, however, still exhibited abundant VAChT-positive C-boutons (red), with the vast majority of them not displaying any association with the scarce Y172-positive profiles remaining. VAChT-positive C-boutons delimited with dotted-line squares in (**F3**; indicated as 1 and 2) are shown in **(G1–G3)** (1) and **(H1–H3)** (2); C-bouton number 1 was associated with a small remnant Y172-positive signal, whereas, Y172-immunoreactivity was completely lost in C-bouton number 2. Scale bars: **D4,E4** = 20 μm (valid for **D1–D3,E1–E3**, respectively); **F4** = 10 μm (valid for **F1–F3**); **H3** = 1 μm (valid for **G1–H2**).

SOD1^G93A^ mice exhibit the first clinical symptoms of disease on approximately P90 and die by approximately P120 (Turner and Talbot, 2008). In these animals, a gradual decrease in the density of peripheral Y172-positive profiles was found in MNs as the disease progressed (Figures 9A,D1–H3). Compared to that in age-matched WT mice, the number of Y172-immunoreactive spots was already decreased in MNs from SOD1^G93A^ animals on P60, well before symptom onset. Interestingly, at this age in the mutant mice, apparently healthy MNs exhibiting abundant cytoplasmic Y172-positive profiles were located in the vicinity of degenerating MNs surrounded by IBA1-positive microglia (Figures 9D1–D4); these degenerating MNs displayed high nuclear Y172 immunoreactivity and very scarce cytoplasmic Y172-immunostained profiles. A more dramatic depletion of Y172 immunolabeling was observed in SOD1^G93A^ MNs on P90 and, particularly, on P120 (~50% and 60%, respectively). Again, at these time points, SOD1^G93A^ MNs that were degenerating and showed intense Y172 immunoreactivity in their nuclei were those that exhibited scarce immunoreactive cytoplasmic profiles; these MNs alternated with MNs that displayed apparently healthy morphology and profuse cytoplasmic Y172 immunolabeling. As expected from our previous analysis (Gallart-Palau et al., 2014), the density of VAChT-immunostained C-boutons in SOD1^G93A^ MNs on P120 was considerably reduced (~35% reduction) compared to that in age-matched WT MNs (Figure 9B); this C-bouton loss in MNs from mutant animals was not noticed on either P60 or P90 (not shown) despite the depletion in Y172-positive profiles found at both time-points of the disease. The number of VAChT-positive puncta that showed a spatial association with the Y172 signal was then quantified on P120. At this age, we found that there was a significant decrease (~45%) in the proportion of SOD1^G93A^ MNs exhibiting VAChT/Y172-positive puncta, compared to that found in WT MNs (Figures 9C,F1–H3).

**Figure 10:**
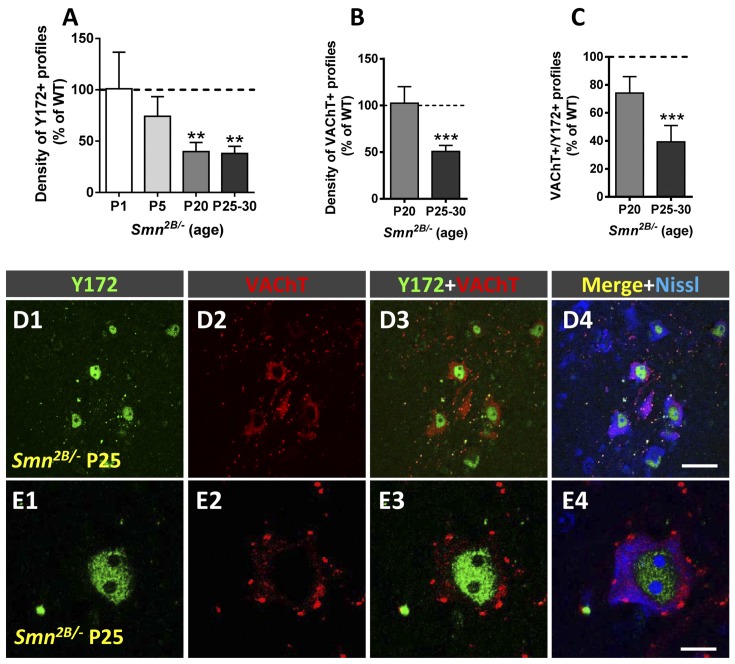
Changes in Y172 immunoreactivity in MNs from *Smn*^2B/-^ mice. **(A–C)** The density (per 100 μm of cell body perimeter) of peripherally located Y172-positive profiles **(A)** and of VAChT-positive puncta **(B)**, and the percentage of VAChT-positive puncta spatially associated with Y172 immunoreactivity **(C)** in mutant mouse MNs at different stages of disease (early presymptomatic [P1 and P5], late symptomatic [P20], and end-stage [P25–30]). **(D1–E4)** Representative confocal micrographs of spinal cord sections from *Smn*^2B/-^ mice at P25 immunostained with Y172 (green) and anti-VAChT (for C-boutons, red) antibodies, as indicated in the panels; sections were also counterstained with fluorescent Nissl stain (blue in **D4** and** E4**) for MN visualization. Note the intense nuclear Y172 immunostaining in **(D1,E1)** and the marked depletion in Y172-positive profiles found in spinal muscular atrophy (SMA) MNs.The data are expressed as the mean ± SEM; 15–20 MNs randomly selected from *Smn*^2B/-^ mice and respective WT littermates were analyzed; **p < 0.01 and ***p < 0.001 vs. WT, student’s *t*-test.Scale bars: **D4** = 40 μm (valid for **D1–D3**); and **E4** = 10 μm (valid for **E1–E3**).

Similar results were obtained in an intermediate model of SMA, the *Smn*^2B/-^ mouse. *Smn*^2B/-^ mice develop a moderately severe form of SMA and exhibit phenotypic alterations starting at ~P12 and a mean lifespan of ~3–4 weeks (Eshraghi et al., 2016; Cerveró et al., 2018). Compared to that in WT littermates, the density of peripheral Y172-positive profiles was already slightly reduced in MNs from *Smn*^2B/-^ mice on P5 (Figure 10A), an age at which significant MN death has been described in this mouse model (Cerveró et al., 2018). The depletion of Y172-positive profiles in *Smn*^2B/-^ MNs was more pronounced at advanced stages of disease (P20-P30; Figures 10A,D1–E4), coinciding with the loss of ~75% of the number of MNs present in WT mice (Cerveró et al., 2018). In contrast, the density of VAChT-positive puncta in *Smn*^2B/-^ MNs was not noticeably changed until the end stage of disease (P25–30; Figures 10B,D1–E4; see also Cerveró et al., 2018), when we also observed significantly less C-boutons with a spatial relationship with peripheral Y172-positive spots (Figure 10C).

### The Specificity of Synaptically Associated p-c-Jun-Like Immunoreactivity Revealed by the Y172 Antibody

**Figure 11:**
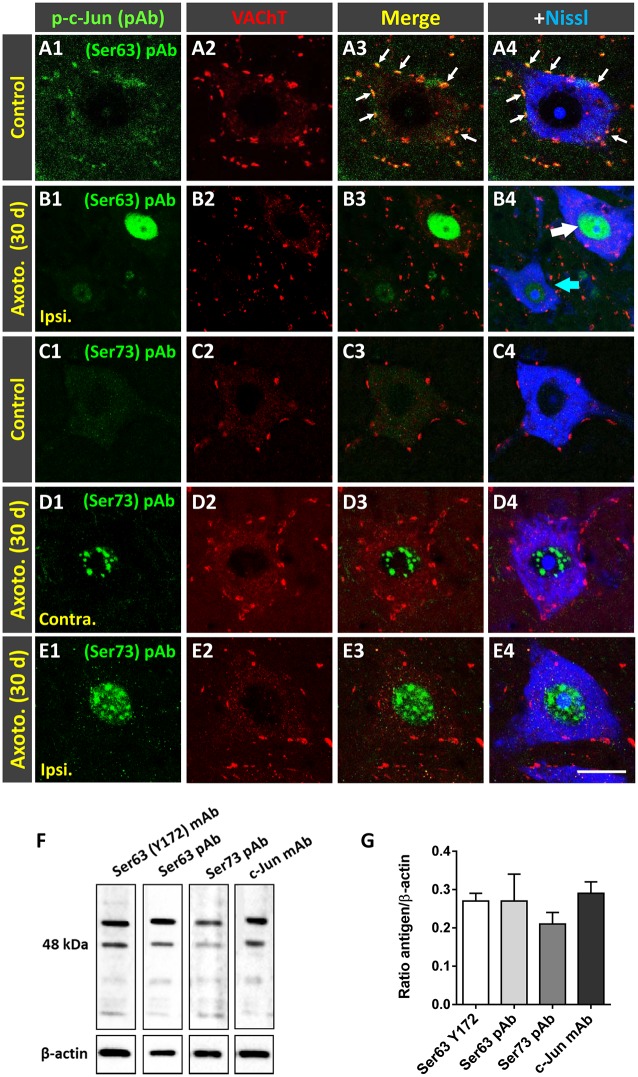
p-c-Jun-like immunodetection with antibodies other than Y172 under basal conditions and in axotomized MNs (30 days after sciatic nerve transection at P60). **(A1–E4)** Representative images of spinal cord MNs double immunostained with different polyclonal antibodies (pAbs) against c-Jun (green) phosphorylated at either Ser63 **(A1–B4)** or Ser73 **(C1–E4)** and VAChT (red); fluorescent Nissl stain (blue in **A4,B4,C4,D4**, and **E4**) was used to visualize MNs. The images in **(A1,B1)**, which show the p-c-Jun (Ser63) channel, were obtained following the modification of scanning parameters to achieve a higher sensitivity of detection than that used for the Y172 antibody. Note the absence of p-c-Jun (Ser63) nuclear immunostaining and the presence of immunolabeled cytoplasmic profiles mainly located in the periphery of the cell body (arrows in **A3,A4**) under basal conditions **(A1–A4)**. Thirty days after unilateral sciatic nerve transection **(B1–B4)**, the same antibody revealed prominent nuclear immunostaining in MNs located on the ipsilateral (operated) side of the spinal cord (white arrow in **B4**) but an almost complete absence of cytoplasmic positive profiles. The intense immunoreactivity in the nucleus of the presumably axotomized MN was in contrast with the faint nuclear immunostaining in the neighboring neuron, likely corresponding to a nonlesioned MN (blue arrow in B4). **(C1–C4)** Negative immunostaining with the polyclonal antibody against p-c-Jun (Ser73) was observed in both the nucleus and cytoplasm of an MN under basal conditions. **(D1–E4)** The same antibody showed intense positive nuclear immunostaining and the absence of immunostained cytoplasmic profiles in axotomized MNs, both in the ipsilateral and contralateral side of the spinal cord. **(F)** Representative western blots of mouse spinal cord extracts probed with the antibodies against p-c-Jun used in our study; a monoclonal antibody against c-Jun (c-Jun mAb) was also included, and β-actin was used as a loading control. Note that a band corresponding to ~43–48 kDa, as expected for p-c-Jun, was observed; the same band was also seen in western blots performed with the anti-c-Jun antibody. **(G)** Densitometric analysis of p-c-Jun bands obtained in western blots by probing with the antibodies used for analysis. The data were normalized to β-actin; the bars represent the values (mean ± SEM) of extracts from three mice. Scale bar: **E4** = 20 μm (valid for **A1–E3**).

Due to the unexpected close association between VAChT-containing synapses and the phosphorylated transcription factor c-Jun found with the Y172 antibody, we decided to analyze whether reproducible results could be obtained with other distinct anti-p-c-Jun antibodies. Two rabbit polyclonal antibodies against c-Jun phosphorylated either at Ser63 or at Ser73 were tested in spinal cord sections from adult mice under basal conditions and after the induction of MN stress, such as that provoked by peripheral nerve axotomy. For the latter condition, samples from mice were examined 30 days after sciatic nerve transection. Under basal conditions, the immunofluorescence obtained with the polyclonal antibody against p-c-Jun (Ser63) was hardly visible when confocal images were captured using the same scanning parameters as those utilized for the Y172 antibody. However, when the microscopy settings were modified to attain a higher sensitivity for detection, the polyclonal antibody exhibited an immunocytochemical pattern similar to that obtained with the Y172 antibody (Figures 11A1–A4). As expected for p-c-Jun, in sections from the lumbar spinal cord of axotomized mice, strong nuclear immunoreactivity was found in most MNs (Figures 11B1–B4). The cytoplasmic spot pattern of immunolabeling found with the Y172 antibody under basal conditions was barely visible with the rabbit polyclonal anti-p-c-Jun (Ser63) antibody in axotomized animals, even after detector optimization (Figures 11B1–B4). This result is consistent with the progressive loss of cytoplasmic p-c-Jun (Ser63) expression observed in axotomized MNs with the Y172 antibody. In contrast with the findings observed with the polyclonal anti-p-c-Jun (Ser63) antibody, the antibody raised against p-c-Jun (Ser73) displayed negative nuclear and cytoplasmic immunostaining under basal conditions (Figures 11C1–C4). However, this antibody showed intense immunoreactivity in the nucleus of axotomized MNs (Figures 11D1–D4). Overall, these results indicate that synaptically associated p-c-Jun-like immunoreactivity is a property shared only by Ser63 phosphospecific antibodies. This raises the question of whether the antigen detected by these antibodies belongs to an unknown protein that shares a common phosphospecific epitope with c-Jun.

We next examined whether the Y172 antibody is able to detect proteins other than p-c-Jun (Ser63) in spinal cord extracts by western blot analysis. We compared the protein band pattern detected by the Y172 antibody with that identified with the other abovementioned antibodies against p-c-Jun and, also, against c-Jun (Figures 11F,G). The Y172 antibody detected a protein ~43–48 kDa in size, as expected for p-c-Jun. This band was also observed with the polyclonal antibodies against p-c-Jun (Ser63) and p-c-Jun (Ser73). A band with a similar molecular weight was also recognized by the polyclonal anti-c-Jun antibody. The obvious similarities between the western blot patterns obtained with the distinct antibodies used indicate that the differences in the cellular pattern (i.e., C-bouton-associated immunostaining) observed with the Y172 antibody do not correlate with any detectable differential western blot band pattern. As it appears to be highly implausible that a transcription factor such as c-Jun plays a role as a synaptically associated protein, the absence of a band other than that for c-Jun in the western blots may be due to the low levels of the Y172 synaptic antigen in spinal cord extracts.

### Y172-Immunolabeling in NSC-34 Cells and Cultured MNs

We next analyzed whether Y172 immunoreactivity could also be observed in *in vitro* models such as NSC-34 MN-like cells and cultured CD1 MNs. Due to the highly restrictive immunostaining found with the Y172 antibody in MNs *in vivo*, a similar pattern of immunolabeling *in vitro* could provide us enough protein for purification and further analyses.

**Figure 12:**
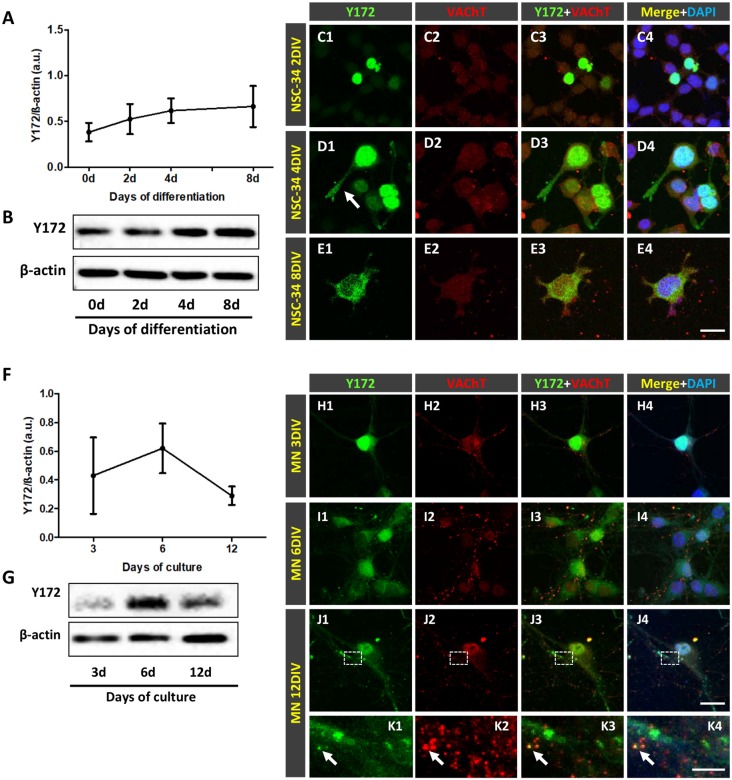
Western blot and immunocytochemical analyses with the Y172 antibody in NSC-34 cells **(A–E4)** and cultured MNs **(F–K4)**. **(A)** The time course of Y172 antigen expression levels in NSC-34 cells after a different number of days (0, 2, 4 and 8) (d) of differentiation, as determined by western blot analysis; the densitometric analysis was normalized to β-actin, which was used as a loading control. **(B)** Representative western blots of NSC-34 cells probed with the Y172 antibody used for quantification in **(A)**. **(C1–E4)** Representative confocal images of NSC-34 cells after 2 **(C1–C4)**, 4 **(D1–D4)** and 8 **(E1–E4)** days of differentiation stained with Y172 (green) and anti-VAChT (red) antibodies as well as DAPI (blue) for DNA, as indicated. Y172 immunostaining was found exclusively in the nuclei of NSC-34 cells following 2 days of differentiation **(C1–4)**; however, granular immunostaining was observed in the cytoplasm of cell bodies and neurites after 4 days of differentiation **(D1–4)**; after 8 days of differentiation, Y172 immunostaining decreased in the nucleus but markedly increased in the cytoplasm of cell bodies and proximal neurites **(E1–4)**. VAChT immunostaining displayed a diffuse and faintly positive pattern into the cell body cytoplasm, and although some positive puncta appear to be present, these were very scarce and did not have a defined location in NSC-34 cells. **(F)** The time course of Y172 antigen expression levels in MNs cultured for 3, 6 and 12 days, determined by western blot analysis; the densitometric analysis was normalized to β-actin, which was used as a loading control. **(G)** Representative western blots of cultured MNs probed with the Y172 antibody used for quantification in **(F)**. (**H1–K4)** Representative confocal images of MNs cultured for 3 **(H1–4)**, 6 **(I1–4)** and 12 **(J1–4)** days stained with Y172 (green) and anti-VAChT (red) antibodies as well as DAPI (blue) for DNA, as indicated. Note the intense nuclear positivity but the faint and discrete cytoplasmic signal obtained with the Y172 antibody at 3 DIV **(H1–4)**; however, at 6 DIV **(I1–I4)**, an increase in the cytoplasmic immunolabeling, mainly in the form of oval patches in the soma and proximal neurites, was observed. Following 12 days of culture **(J1–4)**, a reduction in Y172 immunolabeling was observed in the cytoplasm, although some positive profiles remained. The area delimited by the dotted rectangle in **(J1–J4)** is shown in **(K1–K4)** at higher magnification; note that some Y172-positive profiles were in close association with patches expressing VAChT (red, arrow). The data in **(A,F)** are shown as the mean ± SEM of 3–6 western blots from three independent cultures. Scale bars: **E4,J4** = 20 μm (valid for **C1-E3,H1–J3**, respectively); **K4** = 4 μm (valid for **K1–K3**).

Differentiated NSC-34 cells resemble mature MNs based on several morphological and physiological features (Cashman et al., 1992; Matusica et al., 2008; Maier et al., 2013). Due to the controversial data reported in the literature concerning the use of RA in NSC-34 cell cultures to achieve greater MN-like phenotypic properties (Johann et al., 2011; Maier et al., 2013; Madji Hounoum et al., 2016), we decided to maintain the cells in differentiation medium (DMEM/Ham’s F12) either containing or not containing RA. After 2–4 days of differentiation, NSC-34 cells exhibited numerous and extensive neurites that appeared more poorly developed in the presence of RA (not shown). This observation is in agreement with a previous study (Madji Hounoum et al., 2016), in which authors showed that the addition of RA to DMEM/Ham’s F12 medium, compared to the same differentiation medium without RA, decreases the average neurite length. Western blot analysis with the Y172 antibody revealed basal levels of p-c-Jun-like protein in undifferentiated cells; at all the time-points studied (after 2, 4 and 8 days of differentiation), the protein expression levels increased in NSC-34 cells cultured without RA but not in those maintained in its presence (Figures 12A,B). Therefore, we chose NSC-34 cells cultured in the absence of RA for our immunocytochemical studies. After 2 days of differentiation, Y172 immunolabeling was located in the nucleus of NSC-34 cells, and there was no detectable positivity into the cell body cytoplasm (Figures 12C1–C4). After 4 days of differentiation, nuclear Y172 immunostaining was reduced, and some immunolabeled cytoplasmic spots were detected (Figures 12D1–D4). After 8 days of differentiation, a drastic decrease in the nuclear Y172 signal, which correlated with an increase in cytoplasmic positive spots, was found in NSC-34 cells (Figures 12E1–E4). Thus, in agreement with our observations in CD1 MNs *in vivo*, NSC-34 cell differentiation resulted in a progressive decrease in nuclear Y172 immunolabeling, which was accompanied by a gradual emergence of positive cytoplasmic spots. To explore whether Y172-immunoreactive spots in differentiated NSC-34 cells display a spatial association with VAChT-positive puncta, double immunofluorescence analysis with Y172 and anti-VAChT antibodies were performed. After 2 days of differentiation, VAChT-immunostaining was already present in NSC-34 cells in the form of a weakly positive diffuse staining distributed in the cytoplasm; no clear and defined concentrations of VAChT-positive puncta were observed in the periphery of cell bodies or proximal dendrites. This pattern of VAChT immunostaining remained unchanged after 4 and 8 days of cell differentiation. This finding is consistent with previous reports (Maier et al., 2013) and indicates the inability of differentiated NSC-34 cells to establish cholinergic synapses. In any case, no consistent spatial association was observed between the Y172-positive and VAChT-positive cytoplasmic spots in differentiated NSC-34 cells on any of the days *in vitro* (DIV) examined.

We next analyzed p-c-Jun-like protein expression with the Y172 antibody in cultured spinal cord MNs. MNs from E13 CD1 mice were collected 3, 6, and 12 days after seeding and processed for immunocytochemistry and western blot analysis (Figures 12F–K4). In MNs cultured for 3 days, prominent nuclear immunostaining was detected in virtually all MNs; conversely, their cytoplasm exhibited faint and diffuse Y172 positivity (Figures 12H1–H4). After 6 DIV, cytoplasmic Y172 immunostaining increased, and several oval patches distributed throughout the soma and proximal dendrites, such as those observed *in vivo* in adult CD1 mice MNs, were seen (Figures 12I1–I4). Unlike those in NSC-34 cells, some Y172-positive patches displayed a close spatial relationship with VAChT-positive puncta, which were distributed in the cell body and neurites of cultured MNs. After 12 DIV, a diminished density of Y172-positive cytoplasmic spots was observed (Figures 12J1–K4). These findings are consistent with the western blot analysis data showing higher levels of p-c-Jun-like protein expression in cultured MNs after 6 DIV and a subsequent moderate decrease at 12 DIV (Figures 12F,G). These results suggest that primary cultured MNs provide an appropriate paradigm to further examine the specificity of Y172 immunostaining.

Because C-bouton-like synapses arise from cholinergic interneurons and because astroglial cells improve MN viability *in vitro*, we also used mixed primary spinal cord cultures from CD1 mice in which both MNs and interneurons were grown on a bed of astroglial cells (Roy et al., 1998). We proposed this type of culture to be more suitable for the development of C-bouton-like synapses arising from cholinergic interneurons, which we believed to be virtually present in these mixed neuronal cultures. MNs were identified by their large size and ChAT immunoreactivity and were maintained as apparently healthy cells for 22–25 DIV. Some MNs showed nuclear Y172 immunofluorescence (Supplementary Figures S4A1–A3), in agreement with the pattern of immunolabeling observed with the antibody *in vitro* in purified MNs, as well as *in vivo* in MNs. In addition, some MNs displayed peripheral Y172-positive profiles similar to those observed *in vitro* in purified MNs and *in vivo* in MNs from the spinal cord (Supplementary Figures S4B1–B3; the number of MNs with Y172-positive peripheral clusters: 12.42% ± 3.05, 12,578 MNs analyzed from 10 cultures maintained for 18–20 DIV). The presence of peripherally located Y172-positive profiles in these MNs was not always associated with nuclear immunolabeling with the Y172 antibody. Interestingly, in contrast to our observations *in vivo*, the vast majority of peripheral Y172-positive profiles in MNs did not appear to be associated with the synaptic marker synaptophysin (Supplementary Figures S4C1–C4); conversely, puncta positive for both Y172 and synaptophysin exhibited a close relationship with VAChT-positive puncta (Supplementary Figures S4D1–D4). Furthermore, electron microscopy examination of cultures using pre-embedding immunolabeling showed clusters of Y172 immunoreactivity at the plasma membrane, frequently without facing synapses (Supplementary Figure S4E). In some cases, however, these Y172-positive profiles were associated with presynaptic, presumably C-type, boutons (Supplementary Figure S4F). Y172 immunoreactivity was also detected in different regions of the MN cytoplasm.

### Downregulation of Y172 Immunostaining in Cultured MNs After a c-Jun Knockdown by RNA Interference

**Figure 13:**
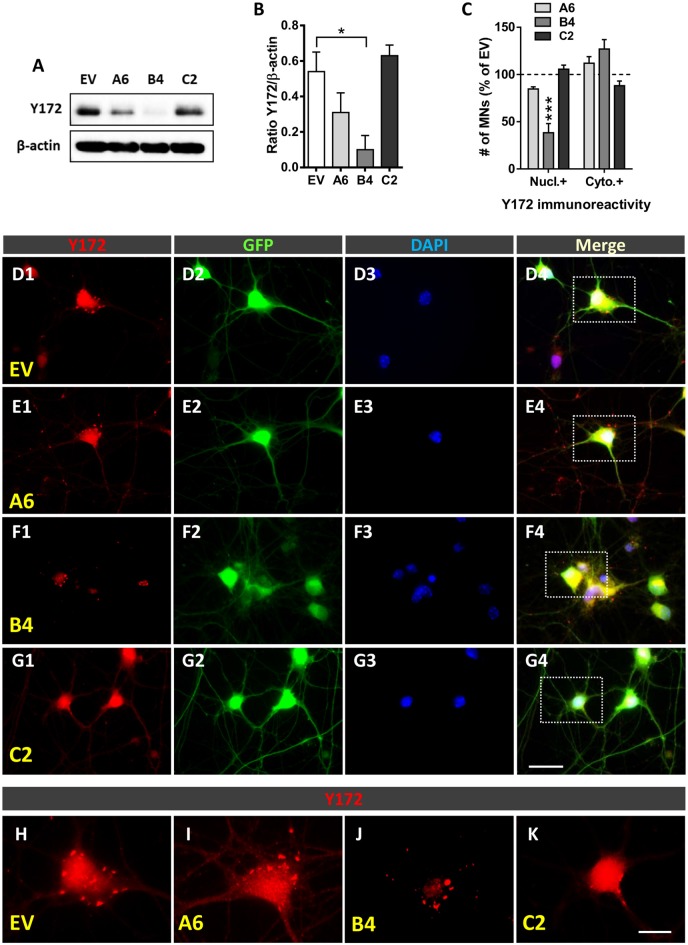
c-Jun knockdown by using shRNAs. **(A)** Representative western blots of cultured MNs transduced with EV, A6, B4, and C2 shRNAs probed with the Y172 antibody; β-actin was used as a loading control. **(B)** Densitometric analysis (expressed as arbitrary units) of western blots probed with the Y172 antibody and normalized to β-actin. The data are shown as the mean ± SEM of four western blots from two independent experiments; *p < 0.05, one-way ANOVA, Bonferroni’s *post hoc* test. **(C)** The number of MNs (expressed as the percentage of EV) exhibiting Y172-positive immunostaining in the nucleus (Nucl.) and cell body cytoplasm (cyto.) of cultured MNs after either A6, B4 or C2 transduction. **(D1–G1)** Representative images of cultured MNs (GFP, green) transduced with either EV **(D1–D4)**, A6 **(E1–E4)**, B4 **(F1–F4)** or C2** (G1–G4)** shRNAs and processed for Y172 immunostaining (red) and DAPI staining (for DNA staining, blue). **(H–K)** High magnification images of the Y172 channel of the areas delimited by dotted-line rectangles in **(D4–G4)** are shown in **(H–K)**, respectively. Note the reduction in nuclear, but not cytoplasmic, Y172 immunoreactivity in the B4-transduced MN shown in **(J)** compared to the non-transduced MN shown in **(H)**. The data in the graphs are expressed as the mean ± SEM; *p < 0.05 and ***p < 0.001 vs. EV, one-way ANOVA, Bonferroni’s *post hoc* test; *n* = 4. Scale bar: **G4** = 20 μm (valid for **D1–G3**); **K** = 10 μm (valid for **H–J**).

RNA interference allows the silencing of gene expression at the posttranscriptional level in a sequence-specific manner and is widely used for the analysis of gene function (Dykxhoorn et al., 2003). We applied this procedure to knock down c-Jun *in vitro* and analyze whether the Y172 antibody indeed detects p-c-Jun or cross-reacts with another protein. For this, we generated three different lentiviral vectors, each one of which contained a different shRNA (A6 shRNA, B4 shRNA, and C2 shRNA, referred to as A6, B4 and C2, respectively) targeting a specific site of the c-Jun mouse sequence and transduced purified cultured MNs. The c-Jun shRNA knockdown efficiency was determined by western blot analysis with the Y172 antibody (Figure 13A). We found that, 6 days after transduction, compared to the EV control, the A6 and B4 shRNAs, but not the C2 shRNA, decreased p-c-Jun-like levels (% reduction: A6, ~40%; B4, ~80%), although the reduction obtained with A6 was not statistically significant (Figure 13B). We next examined Y172 antibody immunofluorescence to determine whether the p-c-Jun-like expression is also reduced in cultured cells after construct transduction. Compared to the percentage in cultures transduced with EV, the percentage of MNs exhibiting p-c-Jun-like-positivity in cultures transduced with shRNA vectors was lower (% decrease compared to EV: A6, ~6%, B4, ~20% and C2, ~8%). We then assessed whether MNs still showing p-c-Jun-like expression following shRNA transduction experience a loss of nuclear or cytoplasmic Y172 immunofluorescence. We found that, compared to EV-transduced cultures, A6- and B4-transduced cultures had lower numbers of cells exhibiting nuclear Y172 positivity (A6, ~15% and B4, ~62%, reduction vs. EV), even though the decrease obtained with A6 was not statistically significant. No changes were observed in C2-transduced cultures (Figures 13C,D1–K). Next, we analyzed whether our shRNA vectors were also able to decrease cytoplasmic Y172 immunolabeling. No reduction in the number of MNs showing cytoplasmic immunostaining was observed for either of the shRNA vectors used. Therefore, B4 was the only shRNA tested that was able to induce a significant reduction in nuclear Y172 immunoreactivity. This result is supported by the finding that B4 was also the most efficient shRNA in decreasing the levels of p-c-Jun-like expression, as detected by western blot analysis with the Y172 antibody. As none of the tested shRNAs against c-Jun appeared to be able to induce a reduction in both nuclear and cytoplasmic Y172 immunoreactivity, we assume that the Y172 antibody detects a synaptic protein other than p-c-Jun.

## Discussion

The C-bouton synapse is presently gaining considerable interest due to recent insights into the knowledge of its complex molecular organization, its association with ER-PM contacts and its involvement in MN pathology (Deardorff et al., 2014; Witts et al., 2014; Salvany et al., 2019). In previous studies, we reported that the NRG1-ErbBs signaling module concentrates at C-boutons and compartmentalizes and associates with different proteins previously known to be expressed in these synapses, such as S1R and Kv2.1 (Gallart-Palau et al., 2014; Casanovas et al., 2017). We noticed that these proteins, along with NRG1, are located in segregated regions inside the SSCs of C-boutons, which apparently represent highly restricted spatial microdomains (Casanovas et al., 2017). We also described that MN activity and peripheral nerve injury induce important changes in the C-bouton organization and provoke the noticeable loss of NRG1 clusters in SSCs (Salvany et al., 2019). Here, we show a novel unidentified molecular component of the C-bouton organization. By means of a monoclonal antibody (clone Y172) against p-c-Jun (Ser63), we described a selective immunocytochemical pattern in adult MNs that differs from what is normally expected for a transcription factor. We found that developing or injured adult MNs displayed strong nuclear Y172 immunostaining that is consistent with the expected nuclear expression of p-c-Jun previously reported under these conditions (Sun et al., 2005; Yuan et al., 2010). In contrast, normal adult MNs showed intense Y172 positivity in structures scattered in the cell body cytoplasm. We noticed that the vast majority of Y172-positive patches peripherally located in the cell body and proximal dendrites were closely associated with C-boutons but not with other types of synaptic afferents projecting to MNs (e.g., glutamatergic or GABAergic inputs). Remarkably, by electron microscopy, it was found that cytoplasmic Y172 immunolabeling was selectively located in SSCs and specific domains of the secretory pathway, particularly in areas of stacked ER cisterns and Golgi-related vesicles. Moreover, MNs from NRG1 type III-overexpressing transgenic mice, in which SSCs are abnormally expanded (Salvany et al., 2019), displayed more abundant and larger Y172-positive profiles than those exhibited by normal MNs, confirming their association with SSC-like organelles. Additionally, we also showed that, in adult mice, peripheral nerve injury results in a noticeable decrease in the density and size of cytoplasmic Y172-positive profiles in axotomized MNs, and that this decrease is dissociated from the time course of C-bouton loss observed under this experimental condition (Salvany et al., 2019). Furthermore, in SOD1^G93A^ and *Smn*^2B/-^ mutant mice, which mimic the human MN diseases ALS and SMA, respectively, degenerating MNs exhibit increased nuclear Y172 immunoreactivity, indicative of c-Jun activation in injured MNs, but a marked depletion in C-bouton-associated Y172-positive profiles, which is consistent with the loss of afferent inputs that occurs in MN diseases (Ling et al., 2010; Mentis et al., 2011; Moreno-López et al., 2011; Sunico et al., 2011; Tarabal et al., 2014; Milan et al., 2015; Cerveró et al., 2018).

Cholinergic synapses and C-boutons barely develop in cultured MNs; we reported here that most of the scarce cholinergic synapses that developed *in vitro* displayed Y172 immunostaining. However, in concordance with our previous data on NRG1 (Casanovas et al., 2017), many MN surface-associated Y172-positive patches were not related to synaptic boutons. These data confirm that the Y172 antigen has an *in vitro* developmental profile analogous to that of other C-bouton-associated proteins.

Our serendipitous finding of the close association between cytoplasmic structures specifically immunolabeled with the Y172 antibody and C-boutons prompted us to analyze whether the antibody crossreacts with an epitope that belongs to a protein other than p-c-Jun. It should be noted that only antibodies recognizing p-c-Jun (Ser63) displayed C-bouton labeling. However, in western blot analyses of spinal cord extracts, we did not find significant differences between p-c-Jun (Ser63) and (Ser73) antibodies. Therefore, we conclude that the synaptic-specific protein detected by the Y172 antibody was not identified by western blot, probably due to its scarce representation in the whole spinal cord extracts used for the analysis. In fact, MNs represent less than 10% of the neuronal population of the spinal cord (Arce et al., 1999), and we estimated that C-boutons do not constitute more than ~4% of total MN afferent synapses. We think that only highly selective sampling procedures, which are beyond the scope of this work, can address the molecular identification of the Y172 binding epitope in MNs. In any case, the Y172 antibody should be considered a new specific marker for C-boutons to be used in future developments. In fact, it is not uncommon that a monoclonal antibody binds to an antigen different from the one that it has been raised against. For instance, the CC1 monoclonal antibody widely used as an oligodendrocyte-specific marker was originally developed against adenomatous polyposis coli (APC), a protein that, oddly, is not present in oligodendrocytes; instead, CC1 binds to the more recently discovered target Quaking 7 in oligodendrocytes (Bin et al., 2016). Another possibility that should not be ignored is that the *c-Jun* gene encodes, by alternative splicing, a yet unidentified tissue-specific protein associated with ER-PM contacts. In this regard, ribbon synapse (RIBEYE), a new identified protein specifically located in retina ribbon synapse, is encoded by the same gene as CtBP2, the *CtBP2/RIBEYE* gene, when an alternative promoter is used. CtBP2 is a transcriptional repressor ubiquitously expressed, whereas RIBEYE is expressed in a tissue-specific fashion and is involved in synaptic vesicle transport and exocytosis (Piatigorsky, 2001). Additionally, the transcription factor fused in sarcoma (FUS), which is normally located in the nucleus, has also been reported to be present at synaptic compartments (Schoen et al., 2016). Nevertheless, in our RNA interference experiments, we were able to significantly reduce Y172 immunostaining in the nucleus but not in the cytoplasm of cultured MNs by using different shRNA vectors targeting specific sites of the c-Jun mouse sequence. This suggests that, with high probability, the Y172 antibody selectively binds an unidentified protein other than p-c-Jun that is located at the postsynaptic compartment of C-boutons. In any case, the results reported here pave the way for future studies aimed at identifying and characterizing the synaptic protein recognized by the Y172 antibody and at further exploring its role in the context of the development, maintenance, plasticity and pathology of C-boutons.

## Data Availability Statement

The raw data supporting the conclusions of this article will be made available by the authors, without undue reservation, to any qualified researcher.

## Ethics Statement

The animal study was reviewed and approved by Committee for Animal Care and Use of University of Lleida.

## Author Contributions

JC and JE conceived and designed the research. AG, PC, OT, AC, SH, SS, LP, RS, JC, and JE performed the research. AG, PC, OT, JC, and JE analyzed the data. JC, JE, OT, and AG wrote the article.

## Conflict of Interest

The authors declare that the research was conducted in the absence of any commercial or financial relationships that could be construed as a potential conflict of interest. The reviewer XN declared a past co-authorship with several of the authors OT, AC, LP, JE, JC to the handling Editor.
