# Supplementary figures and images for: The Y172 Monoclonal Antibody Against p-c-Jun (Ser63) Is a Marker of the Postsynaptic Compartment of C-Type Cholinergic Afferent Synapses on Motoneurons
Source: Front Cell Neurosci. 2020 Jan 24;13:582. doi: 10.3389/fncel.2019.00582 (PMC6992659; doi:10.3389/fncel.2019.00582)

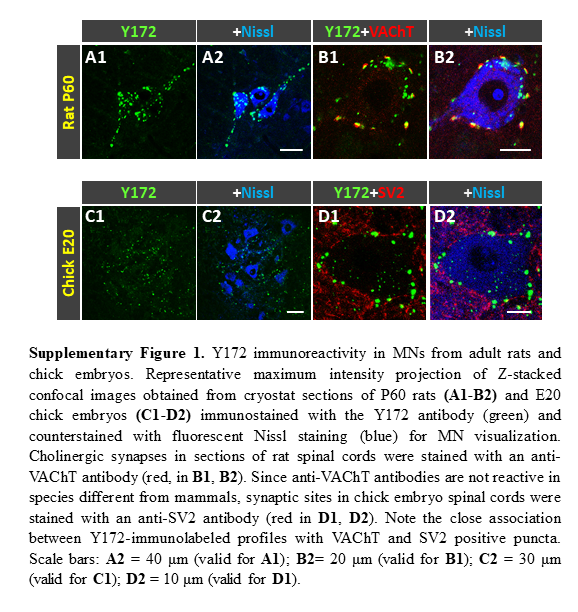

Supplement: Supplementary file 1 [file Image_1.TIF]

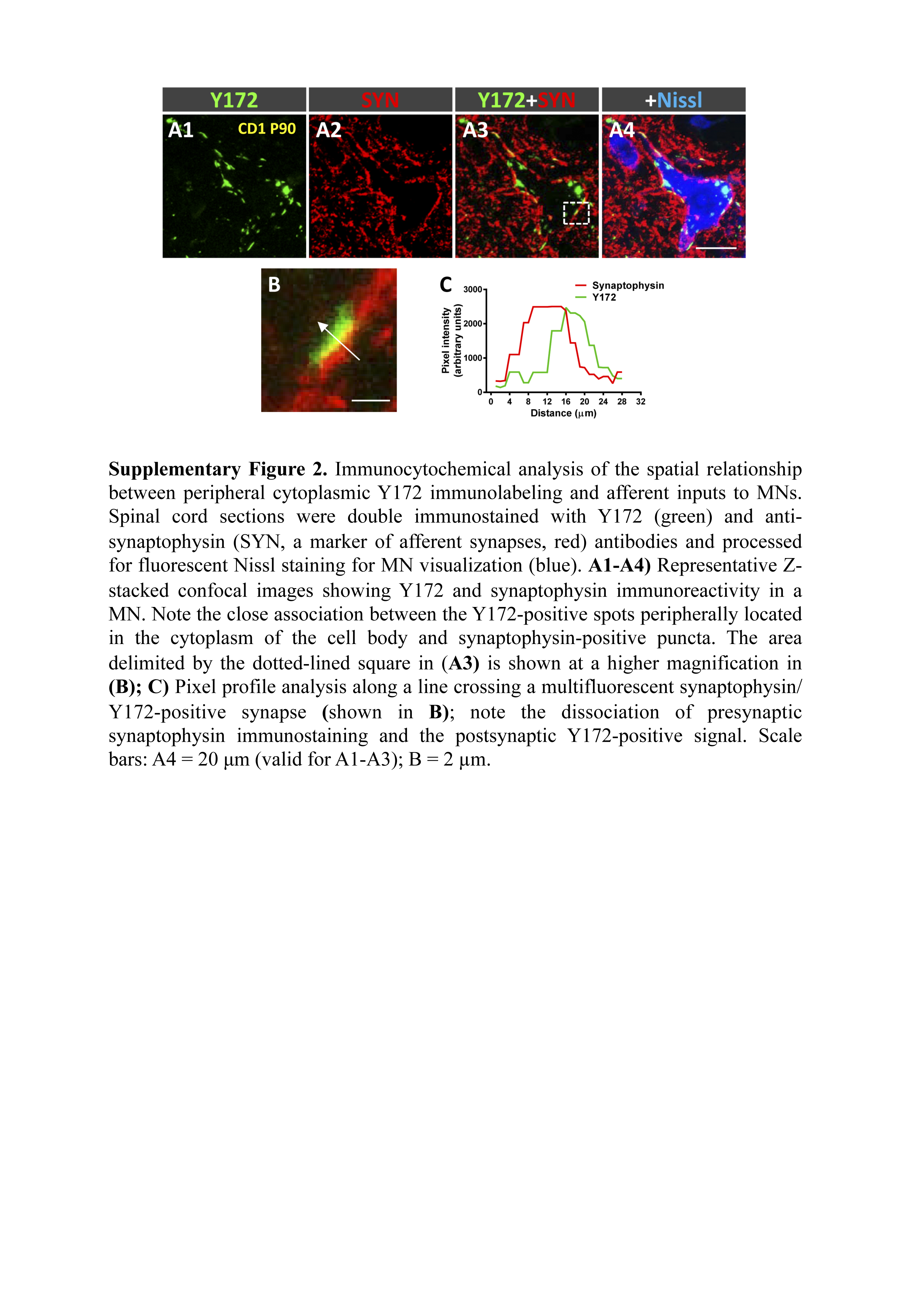

Supplement: Supplementary file 2 [file Image_2.TIF]

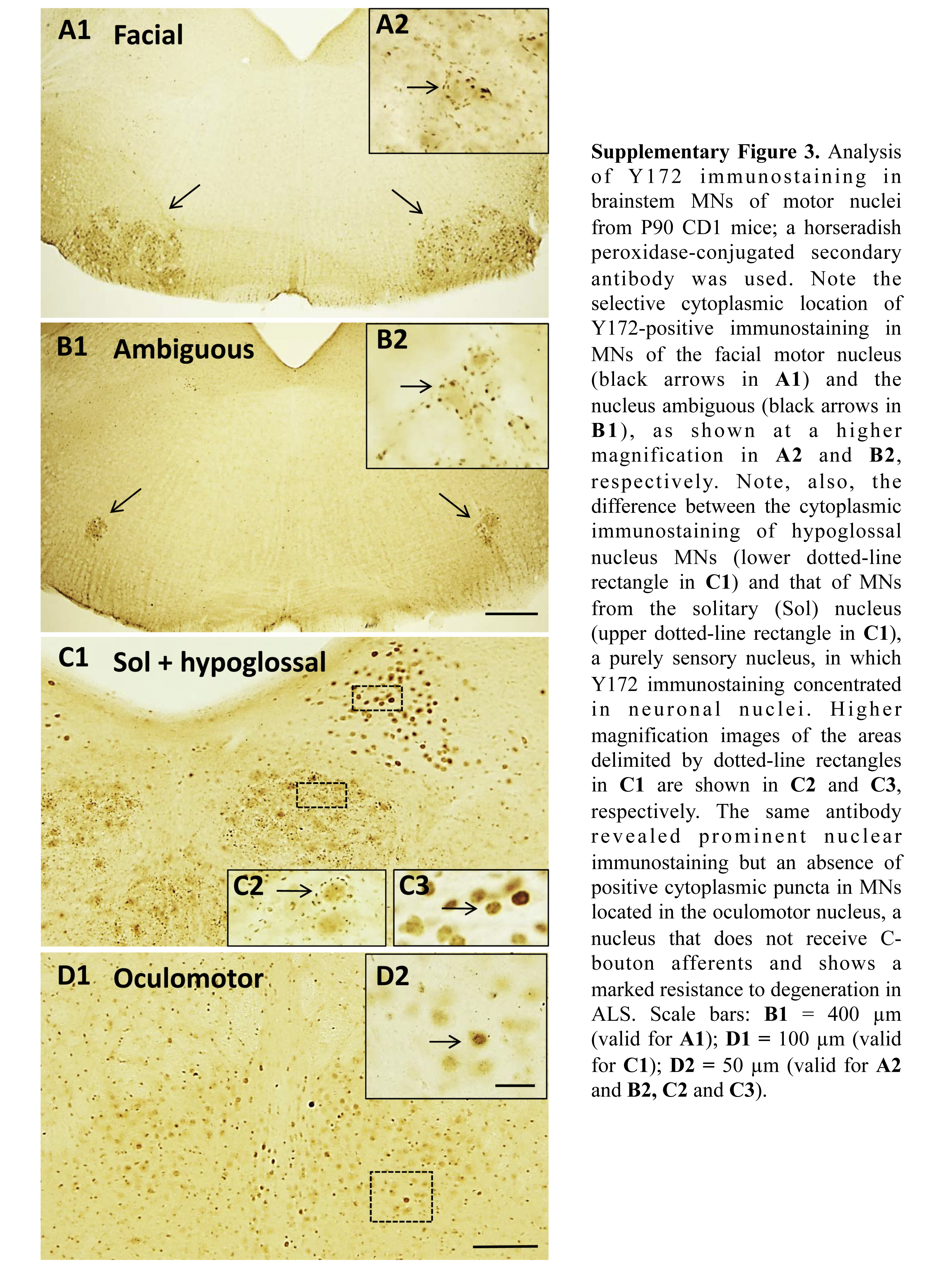

Supplement: Supplementary file 3 [file Image_3.TIF]

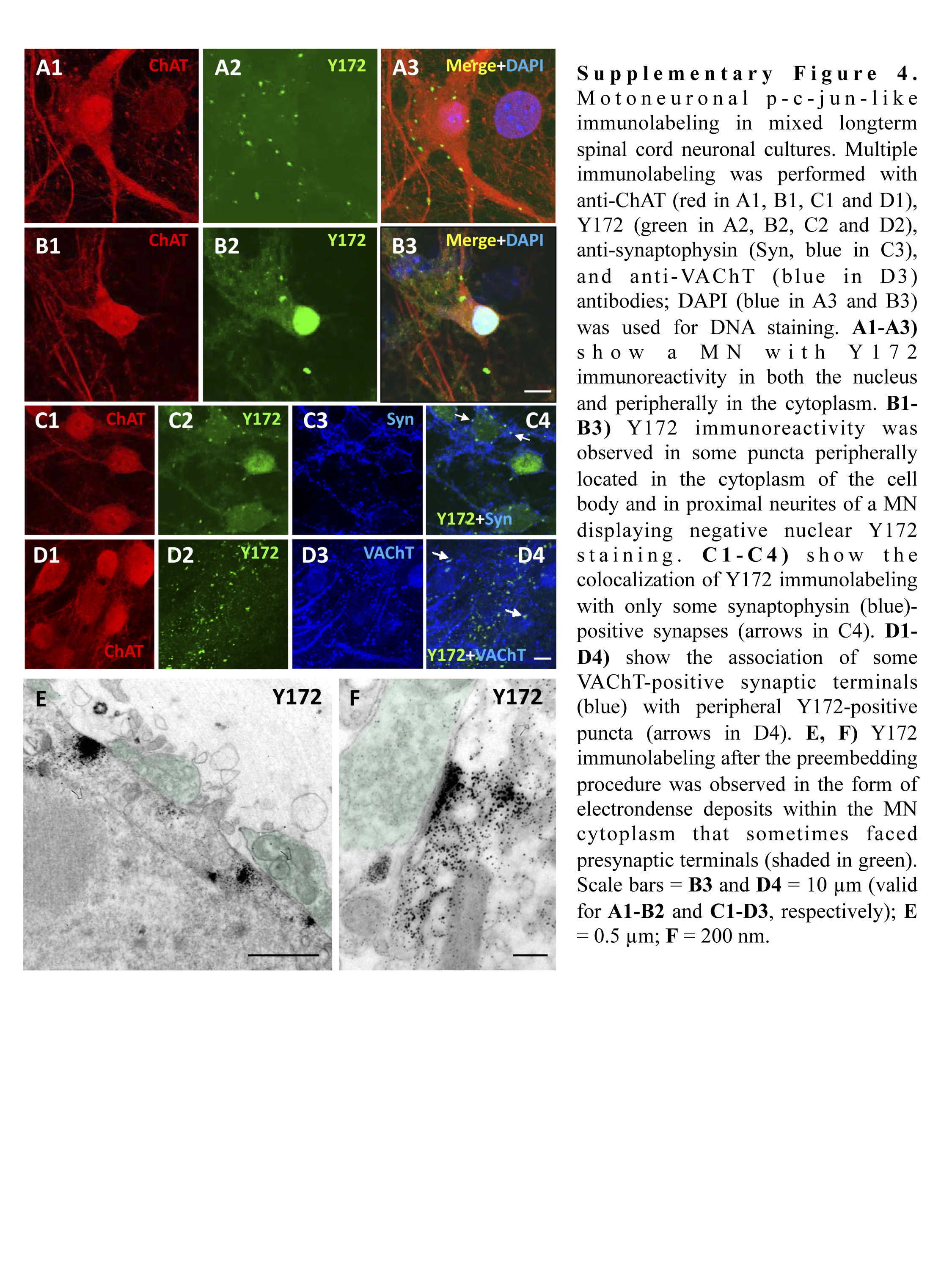

Supplement: Supplementary file 4 [file Image_4.tif]
